# Species Turnover and Environmental Filtering Structure Plant Communities in Moist Temperate Forests

**DOI:** 10.1002/ece3.74054

**Published:** 2026-07-29

**Authors:** Mehreen Samad, Nadeem Ahmad, Adnan Ahmad, Abeer Al‐Andal, Adam Khan, Abdul Rahman Osmani, Muhammad Waheed

**Affiliations:** ^1^ Department of Botany University of Peshawar Peshawar Pakistan; ^2^ Department of Forestry Shaheed Benazir Bhutto University Sheringal Pakistan; ^3^ Department of Biology, College of Science King Khalid University Abha Saudi Arabia; ^4^ Department of Botany University of Lakki Marwat Lakki Marwat Pakistan; ^5^ Department of Zoology Kabul University Kabul Afghanistan; ^6^ Department of Botany, Faculty of Life Sciences University of Okara Okara Pakistan

**Keywords:** community assembly, diversity, environmental gradients, moist temperate forests, species turnover

## Abstract

Montane moist temperate forests are structured by steep elevational and edaphic gradients that drive plant community turnover over short spatial scales. However, integrated evidence linking community structure, diversity, and species turnover to environmental gradients remains limited for the Hindu Kush–Western Himalayan region. This study aimed to characterize plant community organization, quantify alpha and beta diversity, and evaluate vegetation–environment relationships in the moist temperate forests of northern Pakistan. Vegetation was sampled during 2022–2023 across 50 representative stands selected to capture environmental heterogeneity using nested quadrats, and phytosociological attributes were recorded. Soil samples were analyzed for texture and key physicochemical properties. Plant species were grouped into communities, and alpha diversity, beta diversity, and multivariate ordination were applied to examine compositional patterns. A total of 70 plant species were recorded, with herbs and hemicryptophytes representing the dominant growth and life forms. Five distinct plant communities were identified, each defined by significant indicator species. Ordination separated communities clearly, with the first two axes explaining 72.31% of total compositional variation. Alpha diversity was highest in the BRI community and lowest, with greatest dominance, in the QCL community. Beta diversity was driven primarily by species turnover, including complete replacement between high‐ and low‐elevation communities, whereas nestedness was minimal. Communities differed significantly in altitude and soil texture fractions and also varied in pH, electrical conductivity, organic matter, nitrogen, and phosphorus. Community composition was most strongly associated with clay, sand, and slope, while within‐community diversity related mainly to soil texture and nutrient availability. These findings demonstrate that environmental filtering promotes strong species replacement across environmental gradients, underscoring the importance of conserving edaphically and topographically heterogeneous habitats.

## Graphical abstract

Plant biodiversity in moist temperate mountain forests is strongly structured by elevational and edaphic gradients that drive community differentiation over short spatial scales. Using integrated vegetation classification, diversity partitioning, and multivariate analyses, we show that species turnover, rather than nestedness, dominates beta diversity, reflecting strong environmental filtering. Soil texture fractions and nutrient availability emerged as key determinants of community composition and within‐community diversity, highlighting the conservation importance of environmentally heterogeneous forest landscapes.

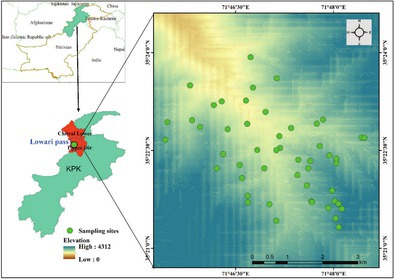

## 1 Introduction

Montane forests span steep environmental gradients along which temperature, moisture availability, and radiation regimes change rapidly (Pepin et al. 2022). These ecosystems play a central role in regulating regional climate, stabilizing slopes, sustaining hydrological cycles, and storing substantial carbon stocks (Sutfin et al. 2016). Recent global assessments show that mountain forests are among the most climate‐sensitive biomes, with warming altering species distributions, forest structure, and ecosystem functioning (Seidl et al. 2020; Thrippleton et al. 2023). Accelerated shifts in snow dynamics and growing‐season length are reshaping regeneration processes and competitive hierarchies, leading to widespread reorganization of forest assemblages (Dollinger et al. 2026). At the same time, geographic isolation and limited upslope space restrict adaptive migration, increasing extinction risk for cold‐adapted taxa (Karim et al. 2025). These pressures place mountain forests at the forefront of ecological research focused on resilience, community reassembly, and long‐term sustainability under changing environmental conditions (Triviño et al. 2023; Murakami et al. 2023).

Plant diversity in forested mountains is strongly influenced by environmental filtering operating across multiple spatial scales (Zheng et al. 2022). Elevation integrates temperature and precipitation regimes, while slope and aspect regulate solar exposure and soil moisture (Liang et al. 2025). These factors interact with soil texture and nutrient availability to influence establishment success, competitive ability, and species dominance (Hoffrén et al. 2022). Contemporary studies increasingly demonstrate that edaphic properties exert a persistent influence on vegetation patterns by shaping water retention and nutrient dynamics (Lira‐Martins et al. 2022). In moist temperate forests, where seasonal moisture is relatively high, subtle variation in soil physical structure can strongly constrain understory composition and tree regeneration (Chelli et al. 2024). Community structure therefore reflects the combined effects of climatic gradients and soil‐mediated resource availability, producing spatially complex mosaics of forest assemblages across mountainous landscapes (Fazlollahi Mohammadi et al. 2022).

Forest biodiversity research has moved beyond species inventories toward integrated frameworks that combine community classification, compositional dissimilarity, and spatial turnover (Storch et al. 2023). Recent advances emphasize the importance of species replacement as a dominant process structuring vegetation across heterogeneous terrain. This mechanism contrasts with simple reductions in species richness (Solomou et al. 2026). High beta diversity driven by turnover is now recognized as a defining feature of mountain ecosystems, reflecting strong habitat specialization and limited dispersal across elevational belts (Ding et al. 2024). Parallel developments in biodiversity mapping enable identification of distinct vegetation units and assessment of their ecological status (Rolls et al. 2023). Such approaches provide insight into how communities respond to environmental gradients and disturbance while supporting conservation prioritization (Pérez‐Toledo et al. 2022). Increasing documentation of upward plant migration and shifts in dominance patterns further underscores the dynamic nature of montane forests and the need for quantitative assessments of community organization (Hiiesalu et al. 2023).

Despite advances in temperate forest ecology, moist temperate forests remain comparatively understudied in terms of integrated assessments that simultaneously examine community classification, diversity patterns, species turnover, and vegetation–environment relationships (Hüttl et al. 2000; Thurner et al. 2017; Landuyt et al. 2019; Stickley and Fraterrigo 2021). Existing research often treats these components separately, limiting mechanistic understanding of forest assembly processes (Weiher et al. 2011; Ali 2019; Sanders and Frago 2024). This study aimed to: (i) characterize plant community structure within the moist temperate forests of Lowari Pass in Upper Dir, northern Pakistan; (ii) quantify diversity patterns and species turnover among delineated communities; and (iii) examine the relationships between vegetation organization and environmental gradients, particularly soil properties and elevation. Based on the theory of environmental filtering, we hypothesized that: (H1) variation in elevation and edaphic conditions would promote the formation of distinct plant communities along the montane gradient; (H2) compositional differences among communities would be driven primarily by species turnover rather than nestedness, reflecting habitat specialization and species replacement across contrasting environments; and (H3) environmentally stressful habitats would support lower diversity and greater dominance by stress‐tolerant species compared with more favorable sites. These hypotheses provide a framework for understanding the mechanisms governing community assembly and biodiversity patterns in moist temperate mountain forests.

## 2 Methodology

### 2.1 Study Area

The moist temperate forests of northern Pakistan are primarily distributed across the Hindu Kush–Western Himalayan ranges, particularly in the Upper Dir and Chitral districts of Khyber Pakhtunkhwa (Khan et al. 2010). These forests typically occur at elevations ranging from approximately 1500 to 3200 m and are characterized by relatively high annual precipitation, cool summers, and cold winters with substantial snowfall. The vegetation is dominated by coniferous and mixed broad‐leaved species, forming structurally complex forest ecosystems that support high floristic diversity. Common canopy‐forming species include *Cedrus deodara* , *Abies pindrow*, *Picea smithiana* , and *Pinus wallichiana* , accompanied by diverse understory shrubs and herbaceous flora. The present study was conducted in the moist temperate forest zone surrounding Lowari Pass, Upper Dir District, located within the Hindu Kush mountain system. Lowari Pass serves as a natural corridor between Dir and Chitral and represents a transitional zone between upper moist temperate and subalpine vegetation belts (Ullah et al. 2015). The study area is characterized by steep slopes, dissected valleys, and rugged terrain, creating strong environmental gradients over short spatial scales. Climatically, the region experiences cold winters with heavy snowfall and mild summers. Forest stands are distributed along varying elevations, aspects, and slopes, producing marked heterogeneity in species composition and community structure. Lower elevations support dense moist temperate coniferous forests, while higher elevations gradually transition toward subalpine communities and alpine grasslands. Following snowmelt, vegetation exhibits rapid growth during the brief summer period (Sajjad et al. 2018).

### 2.2 Field Work and Vegetation Sampling

**FIGURE 1:**
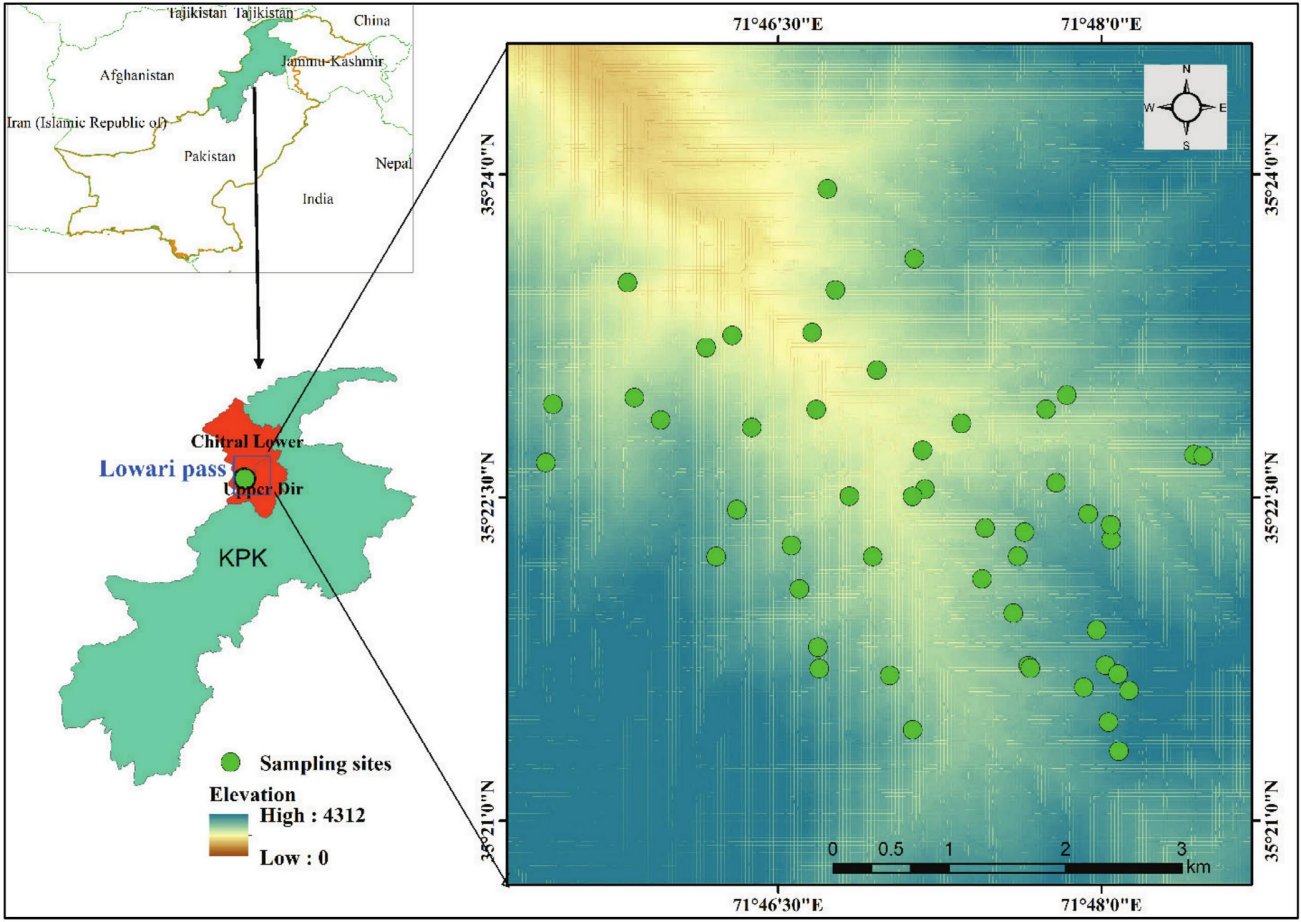
Location of the study area and distribution of sampling stands in the moist temperate forest of Lowari Pass, Upper Dir, northern Pakistan.

Preliminary reconnaissance surveys were conducted in 2022 to identify representative sampling locations. Vegetation and environmental data used in this study were collected during the peak growing season (May–August) of 2023. Prior permission for specimen collection was obtained from the local Forest and Wildlife Department. A total of 50 stands were selected within the moist temperate forest zone near Lowari Pass following reconnaissance surveys to represent the range of habitat conditions, physiognomic structure, altitudinal gradients, and species composition present in the study area (Waheed et al. 2026) (Figure 1). Thus, the sampling design was intended to provide broad coverage of the environmental heterogeneity of the moist temperate forest rather than strict random sampling. The 50 sampling stands were distributed across approximately 12 km^2^ and encompassed the major environmental gradients and vegetation types present within the study area. The distance between sampling stands ranged from 1 to 5 km, reducing the likelihood that adjacent stands represented the same local vegetation patch. Vegetation was sampled using the quadrat method following a nested sampling design. Within each stand, a 20 × 20 m tree quadrat was established. A 10 × 10 m shrub quadrat was nested centrally within the tree quadrat, and five 1 × 1 m herb quadrats were placed within the shrub quadrat, with one located at the center and four at the corners to capture within‐stand variation (Ilyas et al. 2025; Manzoor et al. 2025; Gillani et al. 2025). Herbaceous data from the five sub‐quadrats were averaged to obtain a single representative value per stand, ensuring consistency across vegetation strata (Sadia et al. 2025). Quantitative data for each species were recorded to calculate phytosociological parameters, including frequency, density, cover, and Importance Value Index (IVI) (Waheed et al. 2024, 2025; Sadia et al. 2024). Plant communities were first delineated using hierarchical cluster analysis based on species composition data. Subsequently, Indicator Species Analysis (IndVal) and species importance value indices (IVI) were used to identify characteristic species and assign names to the resulting community groups according to their dominant and ecologically significant taxa. Plant specimens were collected and identified with the assistance of taxonomists using the *Flora of Pakistan* (http://www.efloras.org/flora_page.aspx?flora_id=5). Scientific nomenclature was further verified using the *Plants of the World Online* (POWO) database to ensure taxonomic accuracy. Voucher specimens were pressed, dried, and deposited in the Herbarium of the Department of Botany, University of Peshawar. Life‐history traits, including Raunkiaer life‐form classes, growth forms, and leaf‐size spectra, were assigned to recorded species based on previously published literature (Haq et al. 2022, 2024; Shaheen et al. 2023; Arshad et al. 2024; Waheed and Arshad 2024).

### 2.3 Environmental Variables and Soil Analysis

Topographic variables, including elevation and slope, were recorded for each sampling stand to characterize environmental gradients influencing vegetation patterns (Waheed, Arshad, et al. 2022). Geographic coordinates and elevation were measured using a Global Positioning System (GPS); altitude was verified with a Hague altimeter, and slope angle was determined using an Abney level (Waheed, Haq, et al. 2022). Soil properties were analyzed to assess edaphic factors regulating plant distribution and community structure (Yousaf et al. 2022). Soil samples were collected from the upper 0–20 cm layer using a soil auger. Within each stand, subsamples were obtained from three locations and combined to form a composite sample. Approximately 20–25 g of homogenized soil was placed in labeled polyethylene bags and transported to the laboratory for physico‐chemical analysis (Manan et al. 2020). Soil pH and electrical conductivity (EC) were determined following the method of Sadia et al. (2024). Soil texture was assessed using the hydrometer method (Bouyoucos 1936), and textural classes (sand, silt, and clay) were assigned using the soil texture triangle (Buckman and Brady 1922). Organic matter content was estimated by the Walkley–Black method; calcium carbonate by acid neutralization; and total nitrogen using the Kjeldahl procedure. Available phosphorus was measured following Chapman and Pratt; potassium was determined by flame emission spectroscopy (Roades method); and total soluble salts (TSS) were analyzed according to AOAC standard procedures (Shah et al. 2013).

### 2.4 Data Analysis

Plant community classification was performed using PC‐Ord software through hierarchical cluster analysis combined with Indicator Species Analysis (IndVal) to identify diagnostic species and delineate vegetation groups (MacCune and Mefford 1997). Communities were named according to the three most important indicator species within each cluster, and species with significant indicator values (*p* ≤ 0.05) were considered diagnostic (De Cáceres et al. 2010). All subsequent statistical analyses were conducted in R. Principal Coordinates Analysis (PCoA) was applied to examine compositional relationships among the identified plant communities using the vegan and ape packages. Species contributions to ordination axes were evaluated to identify taxa driving community separation. Alpha diversity indices were calculated community‐wise using the vegan package (Oksanen et al. 2013). Alpha diversity indices were calculated for each sampling stand using the vegan package in R (Oksanen et al. 2013). Community‐wise differences in Shannon diversity, Simpson diversity, dominance, evenness, and environmental variables were tested using one‐way ANOVA/Gaussian models, followed by Tukey‐adjusted post hoc comparisons using the emmeans and multcomp packages. Compact letter displays were used to indicate significant differences among communities at *p* ≤ 0.05. Boxplots were produced with ggplot2, where each point represents one sampling stand, the central line indicates the median, boxes show the interquartile range, and whiskers show data spread excluding outliers (Wickham 2011). Beta diversity among communities was quantified using presence–absence data and partitioned into species turnover (βSIM) and nestedness‐resultant dissimilarity (βSNE) using the betapart package based on the Sørensen family of dissimilarity indices (Baselga and Orme 2012).

Canonical Correspondence Analysis (CCA) was performed using the vegan package to examine associations between plant community composition and measured environmental variables (Oksanen et al. 2013). Before selecting variables for the final model, Pearson correlation analysis was conducted to examine relationships among environmental predictors and to identify potentially redundant variables (Figure S1). A reduced CCA model was then fitted using the selected predictors sand, clay, and slope. Overall model significance, individual environmental predictors, and canonical axes were tested using 999 permutations. To evaluate the relative role of altitude, partial CCA was performed to test whether altitude explained additional compositional variation after accounting for sand, clay, and slope. Variance partitioning was conducted on a Hellinger‐transformed species matrix to separate the pure and shared fractions explained by edaphic and topographic predictor sets. A second variance‐partitioning model was also fitted using soil texture and topographic predictors to quantify the contribution of soil particle‐size gradients more directly. Independent contributions of the retained predictors were further estimated using hierarchical partitioning, with permutation tests used to assess statistical significance. To test whether species turnover among communities exceeded random expectations, beta‐deviation analysis was conducted using presence–absence data. The occurrence matrix was randomized while preserving both site richness and species occupancy, and observed βSIM values were compared with the null distribution using the betapart package (Baselga and Orme 2012). Ordination and summary figures were prepared using ggplot2 and ggrepel (Wickham 2011). Species and site scores were examined to interpret compositional responses along major environmental gradients. Community‐wise relationships between Shannon diversity and environmental variables were explored using second‐order polynomial regression models implemented in the *stats* package. Polynomial functions were employed to examine potential non‐linear responses of diversity to environmental gradients. Given the exploratory nature of the analysis and the relatively small sample size within individual communities, these models were used primarily to identify general patterns rather than to establish predictive relationships. Model fit was evaluated using coefficients of determination (*R* ^2^) and significance levels, and regression visualizations were generated using *ggplot2*. All statistical tests were conducted at a significance level of *p* ≤ 0.05.

## 3 Results

### 3.1 Floristic Composition and Functional Traits

A total of 70 plant species belonging to 64 genera and 33 families were recorded from the moist temperate forest. Dicots dominated the flora with 52 species, followed by monocots (12 species) and gymnosperms (6 species) (Table S1). Asteraceae was the most species‐rich family (8 species), followed by Fabaceae (7), Lamiaceae (6), and Pinaceae (5), while Poaceae and Ranunculaceae each contributed four species. Most remaining families were represented by one or two species. Herbs constituted the largest growth form (40 species), followed by shrubs (21) and trees (9). The life‐form spectrum was dominated by hemicryptophytes (28 species), with therophytes and chamaephytes contributing 10 species each, nanophanerophytes nine species, mesophanerophytes six species, and microphanerophytes five species, whereas geophytes and megaphanerophytes were each represented by a single species.

### 3.2 Classification of Plant Communities

**FIGURE 2:**
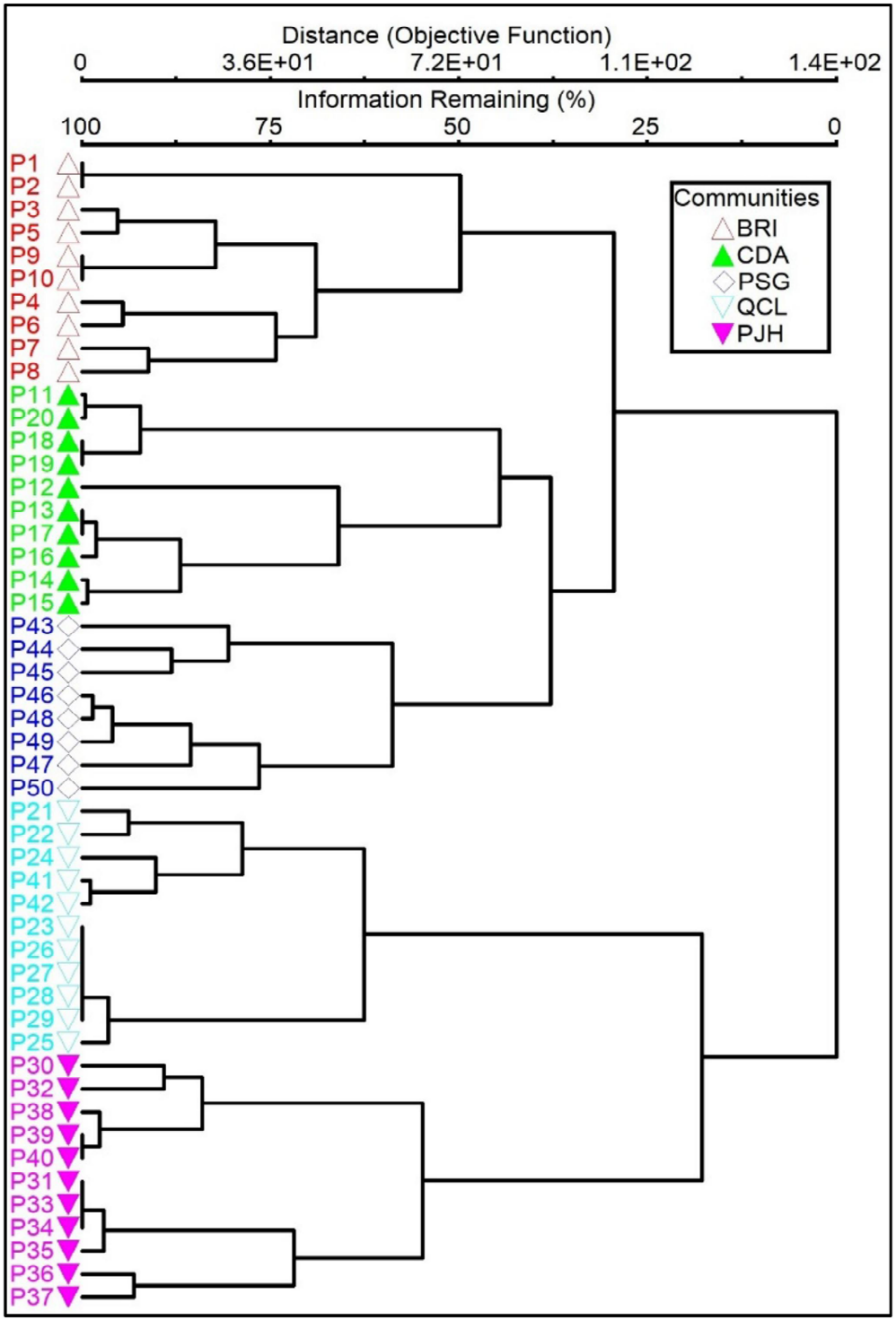
Hierarchical cluster analysis of 50 representative sampling stands showing classification into five plant communities: BRI (*n* = 10), CDA (*n* = 9), PSG (*n* = 10), QCL (*n* = 11), and PJH (*n* = 10).

Hierarchical cluster analysis coupled with Indicator Species Analysis (IndVal) resolved five distinct plant communities across the 50 sampling plots, with communities named after the three most influential indicator species and taxa exhibiting significant indicator values (*p* ≤ 0.05) considered diagnostic (Figure 2). The *Berberis–Rumex–Indigofera* community (BRI) comprised 10 plots and was primarily characterized by *Berberis lycium* (IndVal = 100, *p* = 0.001), followed by *Rumex dentatus* (66.67, *p* = 0.001) and *Indigofera articulata* (36.56, *p* = 0.008), with additional significant contributions from *Lamium album* , *Abies pindrow*, and *Fragaria vesca* , whereas *Trifolium repens* and *Cynodon dactylon* exhibited low, non‐significant associations (Table S2). The *Cedrus–Delphinium–Astragalus* community (CDA), consisting of nine plots, was dominated by *Cedrus deodara* (53.33, *p* = 0.001), *Delphinium nordhagenii* (34.52, *p* = 0.002), and *Astragalus pyrrhotrichus* (26.67, *p* = 0.006), with *Verbascum thapsus* also showing significant fidelity, while remaining taxa including *Schkuhria pinnata* , *Ajuga bracteosa*, *Juniperus indica*, and *Rumex hastatus* displayed weak and non‐significant indicator values. The *Picea–Silene–Galium* community (PSG) encompassed 10 plots and was defined by *Picea smithiana* (62.5, *p* = 0.001), *Silene logisepala* (37.5, *p* = 0.004), and *Galium ghilanicum* (30.18, *p* = 0.006), with additional significant associations from *Delphinium kohatense*, *Daphne mucronata* , *Eremurus stenophyllus*, *Ephedra intermedia*, and *Nerium indicum*, whereas *Calotropis procera* showed non‐significant affinity. The *Quercus–Carex–Lactuca* community (QCL), represented by 11 plots, was strongly structured by *Lactuca virosa* (63.64, *p* = 0.001) and *Quercus baloot*, alongside *Artemisia vulgaris* (45.45, *p* = 0.001), with moderate association of *Aconitum chasmanthum*, while *Avena fatua* , *Pinus wallichiana* , and *Galium ghilanicum* exhibited weak community fidelity. The *Pinus–Justicia–Hedera* community (PJH), comprising 10 plots, was characterized by *Pinus wallichiana* (90, *p* = 0.001), *Justicia adhatoda* (70, *p* = 0.001), and *Hedera nepalensis* (67.99, *p* = 0.001), with strong association of *Ricinus communis* and moderate contributions from *Oxalis corniculata* and *Paeonia officinalis* , whereas *Amaranthus spinosus* and *Cedrus deodara* showed low and non‐significant indicator values.

### 3.3 Relationship Between the Communities

**FIGURE 3:**
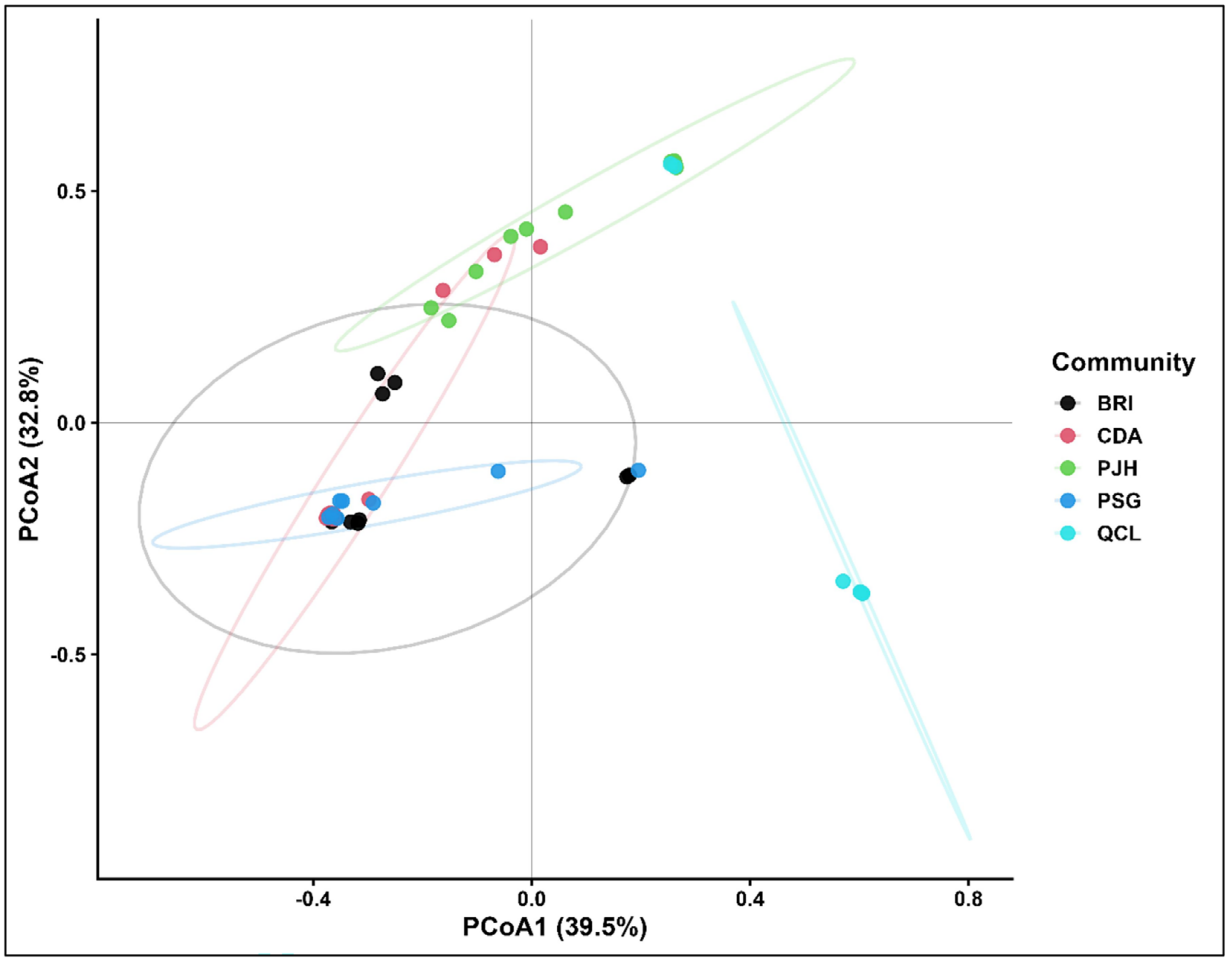
Principal Coordinates Analysis (PCoA) ordination showing compositional relationships among 50 representative sampling stands classified into five plant communities: BRI (*n* = 10), CDA (*n* = 9), PSG (*n* = 10), QCL (*n* = 11), and PJH (*n* = 10). Points represent individual stands and colors indicate community identity.

To investigate the compositional variation of the five identified plant communities, Principal Coordinates Analysis (PCoA) was conducted (Figure 3). The two major coordinate axes described a substantial proportion of the overall variation in species composition. PCoA1 had an eigenvalue of 6.341 and explained 39.5% of the total variation, while PCoA2 had an eigenvalue of 5.267 and explained 32.8% of the variation. Together, the first two axes accounted for 72.3% of the cumulative variation (Table 1). PCoA3 and PCoA4 explained 11.1% and 6.7% of the variation, respectively, bringing the cumulative variation explained by the first four axes to 90.1%. The ordination graph of the first two axes demonstrated that there were spatial clusters of plots of five plant communities (BRI, CDA, PJH, PSG, and QCL). QCL plots were placed at different positions on the positive side of PCoA1, whereas PJH plots were placed on the upper part of PCoA2. BRI and PSG communities were found mostly in the negative direction of PCoA1, and CDA plots were in the middle of both axes.

**TABLE 1:** Eigenvalues and percentage of explained variation for the first five axes of the Principal Coordinates Analysis based on species composition data.

Axis	Eigenvalue	Percent
PCoA1	6.341	39.5
PCoA2	5.267	32.8
PCoA3	1.768	11.1
PCoA4	0.977	6.7
PCoA5	0.389	2.4

### 3.4 Alpha Diversity Patterns Across Communities

**FIGURE 4:**
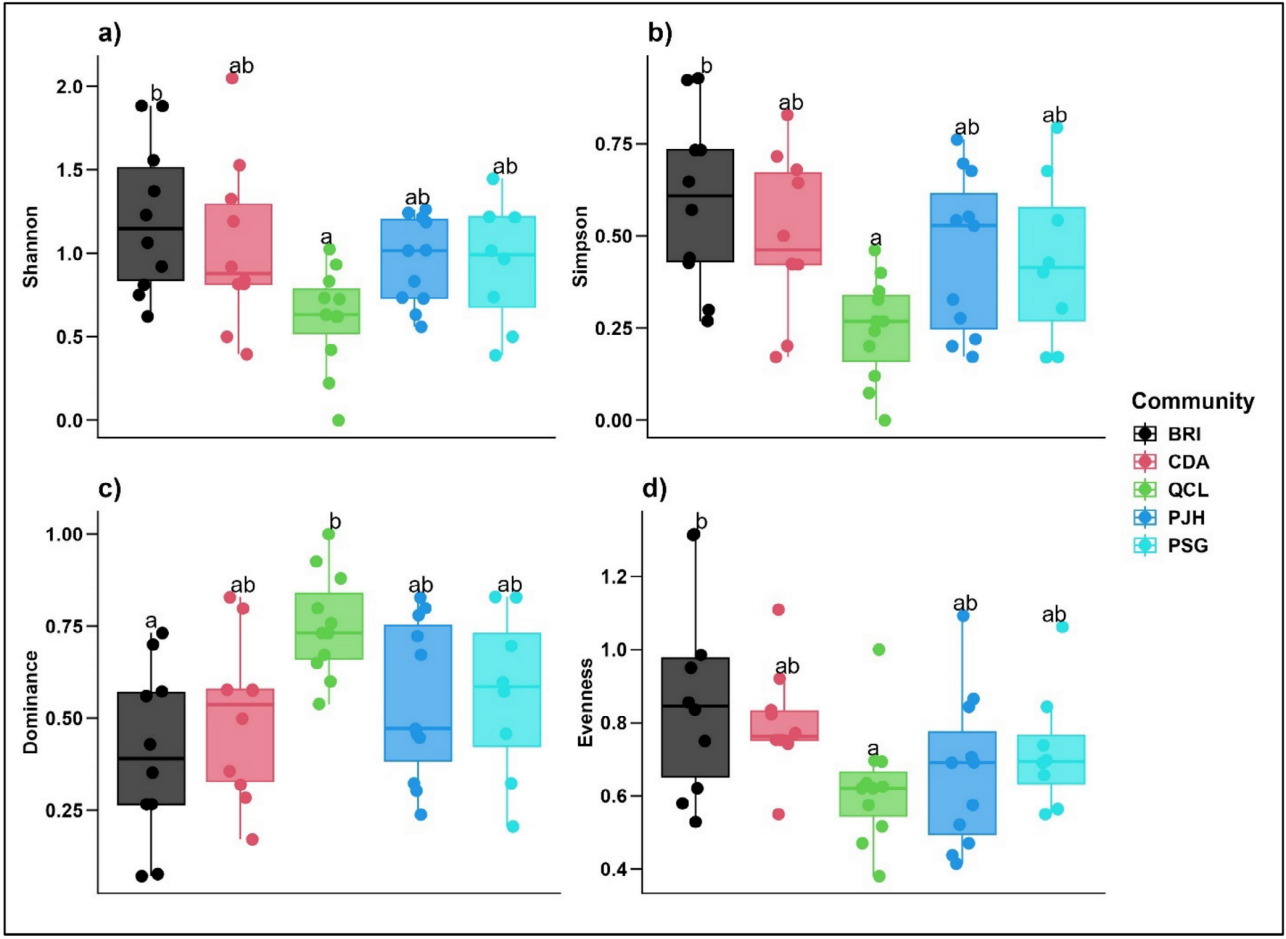
Comparison of alpha diversity indices among five plant communities: (a) Shannon diversity, (b) Simpson diversity, (c) Dominance, and (d) Evenness. Sample sizes were BRI = 10, CDA = 9, QCL = 11, PJH = 10, and PSG = 10 stands. Each point represents one sampling stand. Boxplots show the median, interquartile range, and whiskers indicating data spread. Differences among communities were tested using one‐way ANOVA/Gaussian models followed by Tukey‐adjusted post hoc comparisons. Different lowercase letters indicate significant differences among communities at *p* ≤ 0.05, whereas shared letters indicate non‐significant differences.

Alpha diversity indices varied among the five plant communities (BRI, CDA, PJH, PSG, and QCL). Mean (± SE) Shannon diversity was highest in BRI (1.209 ± 0.144), followed by CDA (1.038 ± 0.157), PJH (0.948 ± 0.079), PSG (0.937 ± 0.131), and lowest in QCL (0.615 ± 0.091) (Table 2). Pairwise comparisons showed a significant difference between BRI and QCL (*p* = 0.008), whereas all other contrasts were not statistically significant. Simpson diversity followed a similar pattern, with the highest mean value in BRI (0.597 ± 0.075), intermediate values in CDA (0.501 ± 0.068), PJH (0.451 ± 0.065), and PSG (0.436 ± 0.080), and the lowest value in QCL (0.247 ± 0.042) (Figure 4). A significant difference was observed between BRI and QCL (*p* = 0.003), while remaining pairwise comparisons were non‐significant (Table S3). Dominance exhibited the highest mean value in QCL (0.753 ± 0.042), followed by PSG (0.564 ± 0.080), PJH (0.549 ± 0.065), CDA (0.499 ± 0.068), and the lowest in BRI (0.403 ± 0.075). Dominance differed significantly between BRI and QCL (*p* = 0.003), whereas other community comparisons did not show significant differences. Evenness was greatest in BRI (0.874 ± 0.088), followed by CDA (0.801 ± 0.045), PSG (0.725 ± 0.058), PJH (0.665 ± 0.063), and QCL (0.621 ± 0.047). A significant difference in evenness was detected between BRI and QCL (*p* = 0.041), while all other pairwise contrasts were statistically non‐significant.

**TABLE 2:** Mean ± SE of alpha diversity indices for each plant community, including Shannon diversity, Simpson diversity, dominance, and evenness. Community sample sizes were BRI (*n* = 10), CDA (*n* = 9), PSG (*n* = 10), QCL (*n* = 11), and PJH (*n* = 10).

Community	Shannon	Simpson	Dominance	Evenness
BRI	1.209 ± 0.144	0.597 ± 0.075	0.403 ± 0.075	0.874 ± 0.088
CDA	1.038 ± 0.157	0.501 ± 0.068	0.499 ± 0.068	0.801 ± 0.045
PJH	0.948 ± 0.079	0.451 ± 0.065	0.549 ± 0.065	0.665 ± 0.063
PSG	0.937 ± 0.131	0.436 ± 0.08	0.564 ± 0.08	0.725 ± 0.058
QCL	0.615 ± 0.091	0.247 ± 0.042	0.753 ± 0.042	0.621 ± 0.047

### 3.5 Beta Diversity Patterns Across Communities

**FIGURE 5:**
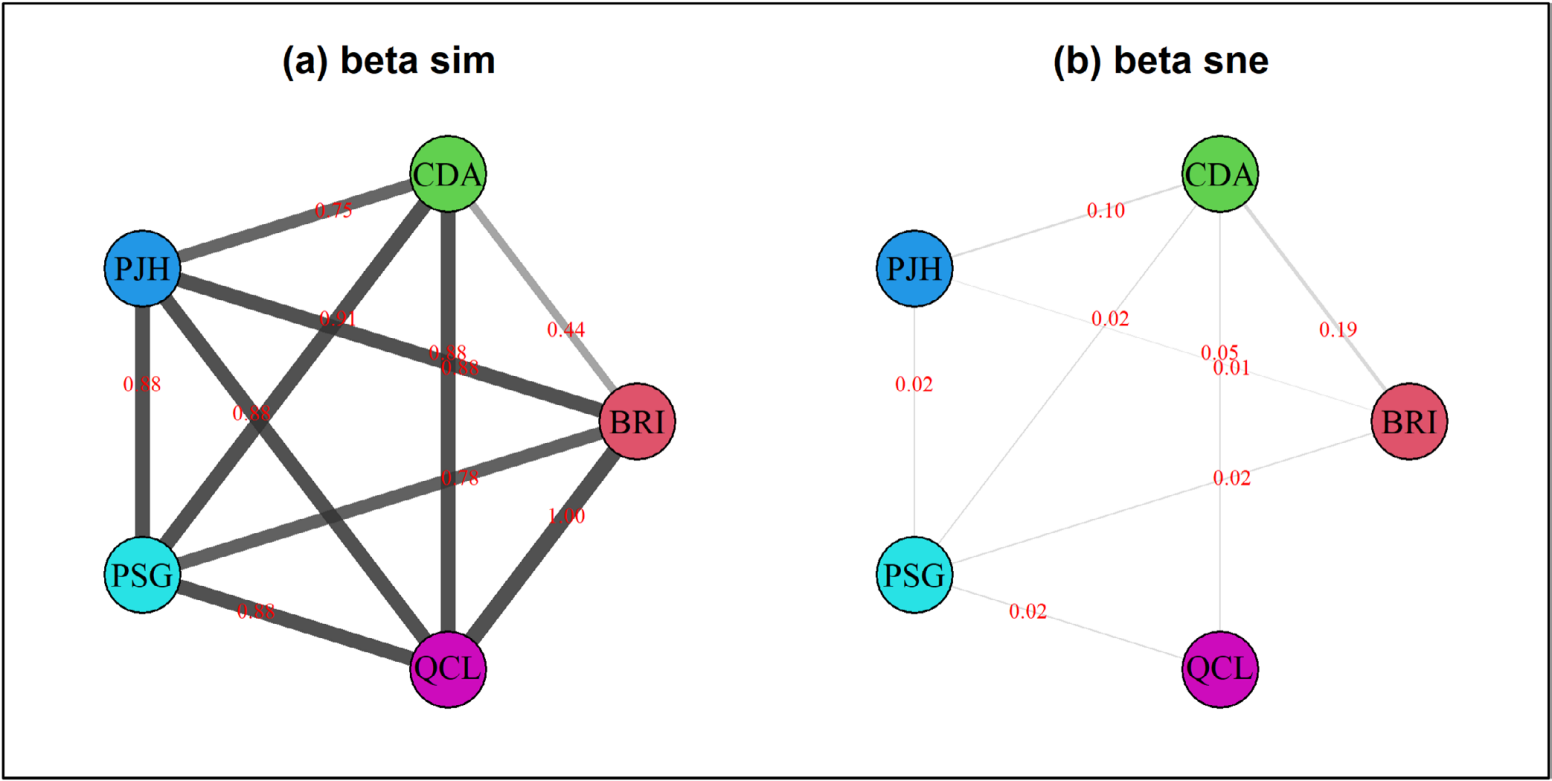
Pairwise beta‐diversity components among the five plant communities: (a) species turnover (βSIM) and (b) nestedness‐resultant dissimilarity (βSNE). Nodes represent communities, edge labels show pairwise values, and edge thickness indicates dissimilarity magnitude. Higher βSIM than βSNE values indicate that beta diversity was mainly driven by species replacement.

Beta‐diversity partitioning showed that compositional dissimilarity among the five plant communities was dominated by species turnover rather than nestedness‐resultant dissimilarity (Figure 5). Pairwise turnover values were high, with βSIM ranging from 0.44 to 1.00. The highest turnover occurred between BRI and QCL (βSIM = 1.00), indicating complete species replacement, whereas the lowest turnover was recorded between BRI and CDA (βSIM = 0.44). Most remaining community pairs showed high turnover values ranging from 0.75 to 0.91. In contrast, nestedness‐resultant dissimilarity was low across all pairwise comparisons, with βSNE ranging from 0.01 to 0.19. The highest nestedness value was observed between BRI and CDA (βSNE = 0.19), while most other community pairs showed very small nestedness contributions. These results confirm that differences among communities were mainly caused by species replacement rather than nested species loss.

### 3.6 Environmental Characteristics of Communities

**FIGURE 6:**
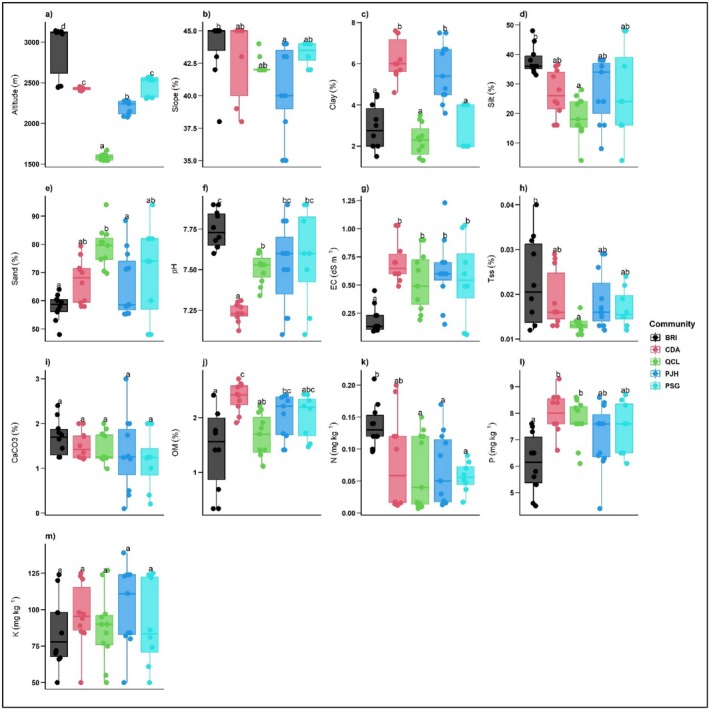
Comparison of topographic and edaphic variables among the five plant communities: (a) altitude, (b) slope, (c) clay, (d) silt, (e) sand, (f) pH, (g) electrical conductivity, (h) total soluble salts, (i) CaCO_3_, (j) organic matter, (k) nitrogen, (l) phosphorus, and (m) potassium. Sample sizes were BRI = 10, CDA = 9, QCL = 11, PJH = 10, and PSG = 10 stands. Each point represents one sampling stand. Boxplots show the median, interquartile range, and whiskers indicating data spread. Community‐wise differences were tested using one‐way ANOVA/Gaussian models followed by Tukey‐adjusted post hoc comparisons. Different lowercase letters indicate significant differences among communities at *p* ≤ 0.05, whereas shared letters indicate non‐significant differences.

Environmental variables differed among the five plant communities (BRI, CDA, QCL, PJH, and PSG), with significant variation detected for several soil and topographic parameters (Figure 6). Mean altitude was highest in BRI (2920.90 ± 102.82 m), followed by PSG (2463.10 ± 43.55 m), CDA (2427.66 ± 5.90 m), PJH (2195.80 ± 25.81 m), and lowest in QCL (1584.77 ± 12.24 m) (Table 3). Pairwise comparisons revealed significant differences among most communities. BRI occurred at significantly higher elevations than CDA (*p* < 0.001), QCL (*p* < 0.001), PJH (*p* < 0.001), and PSG (*p* < 0.001). QCL was significantly lower than PJH and PSG (*p* < 0.001), and PJH differed significantly from PSG (*p* = 0.006). Slope also varied among communities, with the highest mean values recorded in PJH (10.545 ± 1.030) and BRI (10.000 ± 0.727), and the lowest values in QCL (2.273 ± 0.190) and PSG (3.250 ± 0.313). Clay content was highest in CDA (6.22% ± 0.32%) and PJH (5.61% ± 0.43%), and lowest in QCL (2.27% ± 0.24%). Significant differences were observed between BRI and CDA (*p* < 0.001), BRI and PJH (*p* < 0.001), CDA and QCL (*p* < 0.001), and PJH and PSG (*p* < 0.001). Silt content was highest in BRI (38.05% ± 1.52%) and lowest in QCL (18.88% ± 2.10%). A significant difference was detected between BRI and QCL (*p* < 0.001). Sand content was highest in QCL (78.85% ± 2.11%) and lowest in BRI (57.77% ± 1.47%). BRI differed significantly from QCL (*p* < 0.001), and QCL differed from PJH (*p* = 0.039) (Table S4).

**TABLE 3:** Mean (± SE) values of altitude and soil physicochemical properties across the five plant communities.

Variable	BRI	CDA	QCL	PJH	PSG
Altitude	2920.9 ± 102.8	2427.7 ± 5.9	1584.8 ± 12.2	2195.8 ± 25.8	2463.1 ± 43.6
Slope	10.0 ± 0.7	4.2 ± 0.9	2.3 ± 0.2	10.5 ± 1.0	3.3 ± 0.3
Clay	2.9 ± 0.3	6.2 ± 0.3	2.3 ± 0.2	5.6 ± 0.4	2.8 ± 0.4
Silt	38.1 ± 1.5	26.8 ± 2.5	18.9 ± 2.1	28.0 ± 3.3	27.0 ± 5.6
Sand	57.8 ± 1.5	67.0 ± 2.5	78.8 ± 2.1	66.2 ± 3.5	70.3 ± 5.9
pH	7.8 ± 0.0	7.2 ± 0.0	7.5 ± 0.0	7.5 ± 0.1	7.6 ± 0.1
EC	0.2 ± 0.0	0.7 ± 0.1	0.5 ± 0.1	0.6 ± 0.1	0.6 ± 0.1
Tss	0.0 ± 0.0	0.0 ± 0.0	0.0 ± 0.0	0.0 ± 0.0	0.0 ± 0.0
CaCO_3_	1.7 ± 0.1	1.5 ± 0.1	1.4 ± 0.1	1.3 ± 0.3	1.2 ± 0.2
OM	1.4 ± 0.2	2.4 ± 0.1	1.7 ± 0.1	2.0 ± 0.1	2.0 ± 0.1
N	0.1 ± 0.0	0.1 ± 0.0	0.1 ± 0.0	0.1 ± 0.0	0.1 ± 0.0
P	6.1 ± 0.4	8.0 ± 0.2	7.7 ± 0.2	7.1 ± 0.4	7.5 ± 0.4
K	84.9 ± 7.7	96.6 ± 7.2	87.6 ± 7.3	102.3 ± 8.3	90.4 ± 10.5

Mean soil pH ranged from 7.24 ± 0.02 in CDA to 7.75 ± 0.04 in BRI. BRI had significantly higher pH than CDA (*p <* 0.001) and QCL (*p* = 0.038). CDA differed significantly from PJH (*p* = 0.007) and PSG (*p* = 0.003). Electrical conductivity was lowest in BRI (0.193 ± 0.038 dS m^−1^) and highest in CDA (0.709 ± 0.06 dS m^−1^). BRI differed significantly from CDA (*p* < 0.001), QCL (*p* = 0.025), PJH (*p* = 0.003), and PSG (*p* = 0.033). TSS was highest in BRI (0.023% ± 0.003%) and lowest in QCL (0.013% ± 0.001%). A significant difference was observed between BRI and QCL (*p* = 0.010). Calcium carbonate ranged from 1.166% ± 0.23% (PSG) to 1.689% ± 0.13% (BRI), with no significant pairwise differences detected among communities. Organic matter content was highest in CDA (2.376% ± 0.084%) and lowest in BRI (1.423% ± 0.233%). Significant differences were recorded between BRI and CDA (*p* < 0.001), BRI and PJH (*p* = 0.037), and CDA and QCL (*p* = 0.009).

Nitrogen content was highest in BRI (0.137% ± 0.011%) and lowest in PSG (0.057% ± 0.008%). BRI differed significantly from QCL (*p* = 0.034), PJH (*p* = 0.046), and PSG (*p* = 0.023). Phosphorus was lowest in BRI (6.12 ± 0.36 mg kg^−1^) and highest in CDA (8.01 ± 0.25 mg kg^−1^). Significant differences were observed between BRI and CDA (*p* = 0.001) and BRI and QCL (*p* = 0.007). Potassium ranged from 84.9 ± 7.74 mg kg^−1^ (BRI) to 102.27 ± 8.30 mg kg^−1^ (PJH), with no significant pairwise differences detected.

### 3.7 Canonical Correspondence Analysis

**FIGURE 7:**
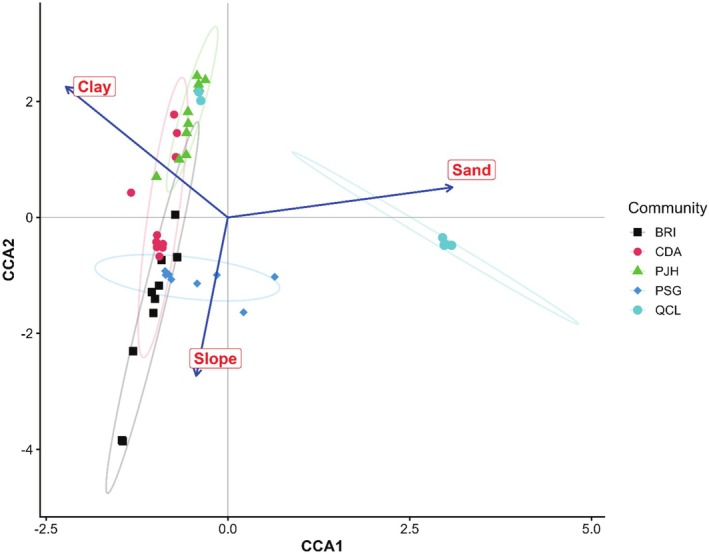
Canonical Correspondence Analysis ordination of sampling stands based on the reduced environmental model. The final model retained sand, clay, and slope as significant predictors of plant community composition. Points represent sampling stands, colors and symbols represent plant communities, ellipses show community‐level dispersion, and arrows show the direction of increasing environmental values.

**FIGURE 8:**
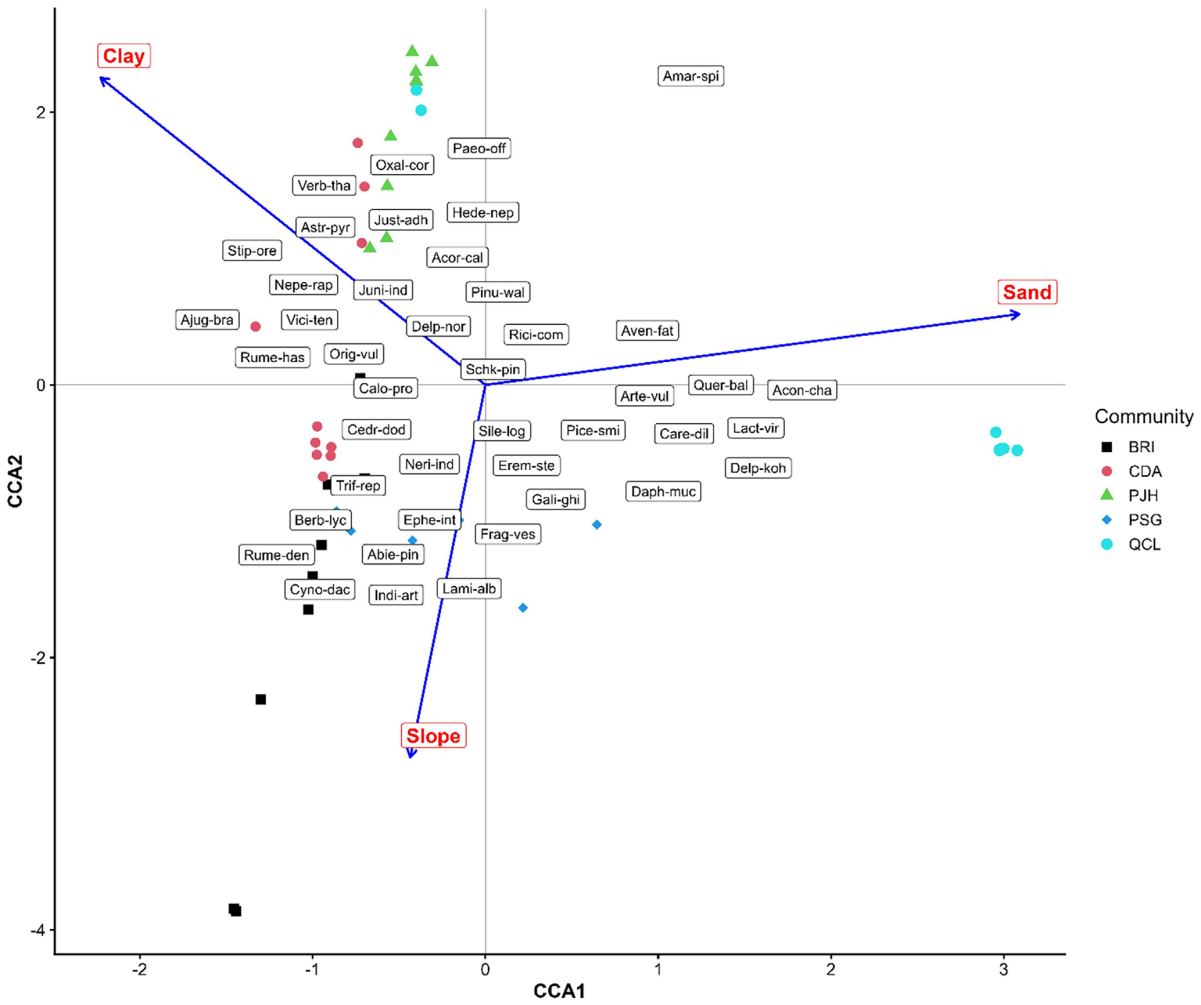
Species‐based Canonical Correspondence Analysis biplot showing the distribution of plant species and sampling stands along the reduced environmental gradients. The ordination is based on sand, clay, and slope. Species abbreviations represent taxa positioned along the first two CCA axes, and arrows show the direction of increasing environmental values.

The CCA retained sand, clay, and slope as final predictors of community composition. The model was significant overall (*χ* ^2^ = 0.867, *F* = 3.346, *p* = 0.001), explaining 17.91% of total compositional variation, with an adjusted *R* ^2^ of 0.126 (Table 4). The first two canonical axes were significant, with CCA1 explaining the largest constrained fraction (eigenvalue = 0.416, *F* = 4.820, *p* = 0.001), followed by CCA2 (eigenvalue = 0.306, *F* = 3.582, *p* = 0.001), whereas CCA3 was non‐significant (*p* = 0.061) (Table 4). Permutation tests confirmed significant associations of community composition with sand (*χ* ^2^ = 0.370, *F* = 4.282, *p* = 0.001), clay (*χ* ^2^ = 0.273, *F* = 3.164, *p* = 0.003), and slope (*χ* ^2^ = 0.224, *F* = 2.593, *p* = 0.009) (Table 5). The ordination separated communities along soil‐texture and terrain gradients, with QCL aligned toward sand, CDA and PJH toward clay, and BRI and part of PSG along the slope‐related gradient (Figure 7). Species distributions showed the same environmental pattern along sand, clay, and slope gradients (Figure 8).

**TABLE 4:** Summary of the reduced CCA model and permutation tests.

Component	df	*χ* ^2^/Eigenvalue	*F*	*p*
Overall CCA model	3	0.867	3.346	0.001
CCA1	1	0.416	4.820	0.001
CCA2	1	0.306	3.582	0.001
CCA3	1	0.145	1.739	0.061
Sand	1	0.370	4.282	0.001
Clay	1	0.273	3.164	0.003
Slope	1	0.224	2.593	0.009
Residual	46	3.971		

**TABLE 5:** Variance partitioning of community composition using Hellinger‐transformed species data.

Predictor set/fraction	Adjusted *R* ^2^	Permutation *p*‐value
*Edaphic + topographic model*		
Full model	0.167	0.002
Pure edaphic fraction	0.092	0.054
Pure topographic fraction	0.063	0.016
Shared edaphic–topographic fraction	0.013	Not testable
Residual variation	0.833	Not testable
*Texture + topographic model*		
Full model	0.204	
Pure texture fraction	0.128	0.001
Pure topographic fraction	0.070	0.002
Shared texture–topographic fraction	0.006	Not testable
Residual variation	0.796	Not testable

Altitude did not explain significant additional compositional variation after accounting for sand, clay, and slope (*χ* ^2^ = 0.044, *F* = 0.505, *p* = 0.714), and it showed no significant independent effect when added to the selected model (*p* = 0.773) (Table S6; Figure S4). Hierarchical partitioning showed that sand had the largest independent contribution, followed by clay and slope, whereas altitude contributed negligibly (Table S6; Figures S3 and S4). Variance partitioning showed that edaphic and topographic variables jointly explained 16.75% of adjusted compositional variation, including 9.19% pure edaphic, 6.31% pure topographic, and 1.25% shared variation (Table S6; Figure S5). In the texture–topography model, soil texture explained a larger pure fraction than topography (12.81% vs. 6.95%), with only 0.61% shared variation and 79.63% residual variation (Table S6; Figure S5). The beta‐deviation null model showed non‐random compositional separation among the delimited communities. Between‐community βSIM was higher than expected under the null model (observed = 0.837, null mean = 0.765, *z* = 8.593, *p* = 0.001), whereas within‐community βSIM was lower than expected (observed = 0.408, null mean = 0.767, *z* = −25.829, *p* = 0.001) (Table S7; Figure S2). The between‐minus‐within βSIM contrast was strongly positive (observed = 0.429, null mean = −0.002, *z* = 31.189, *p* = 0.001), showing that turnover among communities exceeded random expectations after preserving site richness and species occupancy (Table S7; Figure S2).

### 3.8 Species Distribution Along the Environmental Gradient

Species scores on the reduced CCA axes showed clear separation along sand, clay, and slope gradients (Figure 8). Species positioned toward the sand vector included *Aconitum chasmanthum, Delphinium kohatense, Lactuca virosa, Quercus baloot, Carex diluta, Daphne mucronata, Artemisia vulgaris*, and *Picea smithiana* . Species associated with the clay side of the ordination included *Verbascum thapsus, Astragalus pyrrhotrichus, Justicia adhatoda, Hedera nepalensis, Oxalis corniculata, Paeonia officinalis*, and *Pinus wallichiana* . Species positioned along the slope‐related side included *Rumex dentatus, Indigofera articulata, Berberis lycium, Abies pindrow, Lamium album, Cynodon dactylon*, and *Trifolium repens* . Species near the ordination center, including *Cedrus deodara, Calotropis procera, Silene logisepala*, and *Schkuhria pinnata* , showed broader occurrence across the sampled communities.

### 3.9 Diversity–Environment Relationships Within Communities

**FIGURE 9:**
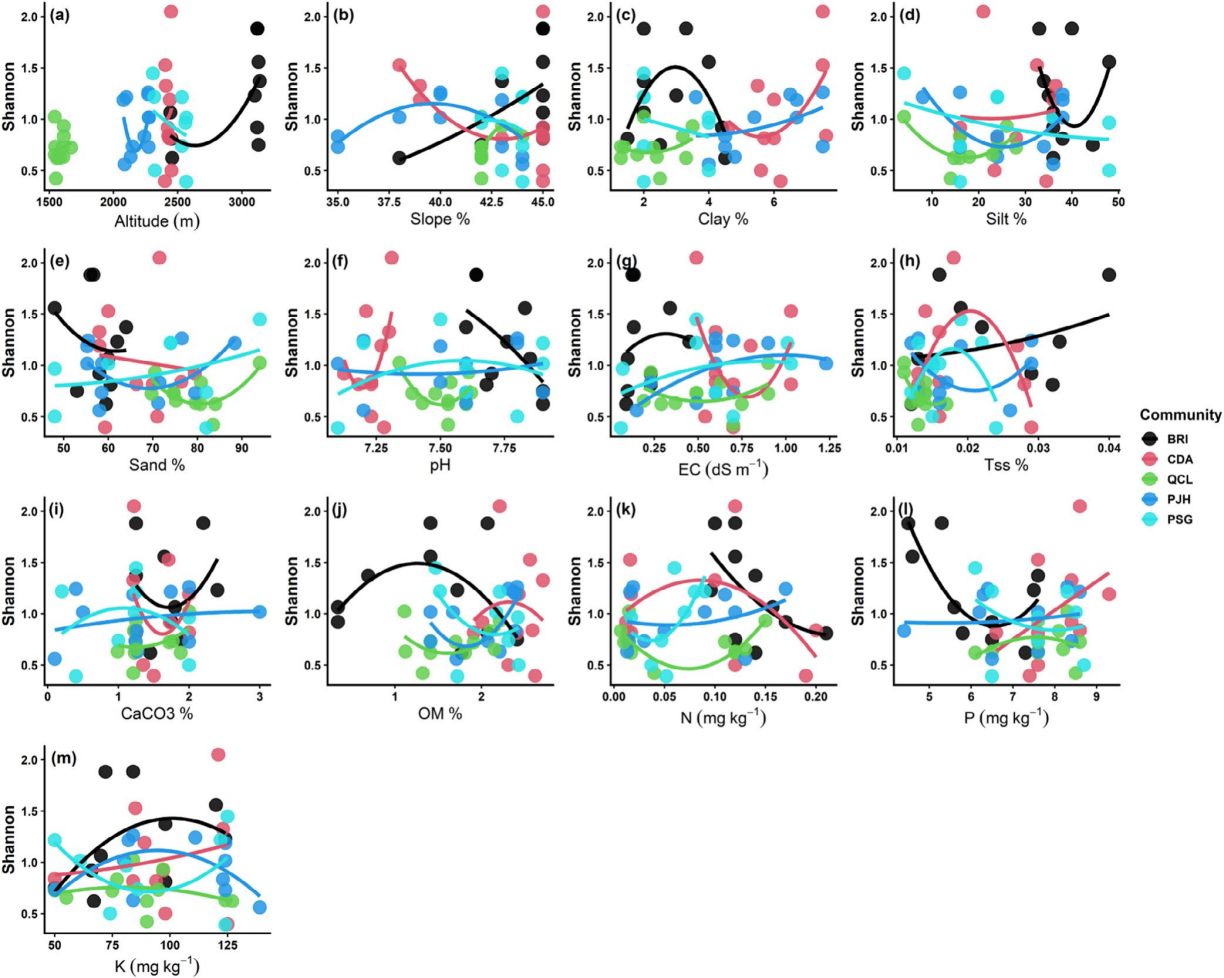
Relationships between Shannon diversity and environmental variables across plant communities. Points represent observed values, while coloured curves depict community‐specific polynomial regression fits for (a) altitude, (b) slope, (c) clay content, (d) silt content, (e) sand content, (f) soil pH, (g) electrical conductivity (EC), (h) total soluble salts (TSS), (i) calcium carbonate (CaCO_3_), (j) organic matter (OM), (k) nitrogen (N), (l) phosphorus (P), and (m) potassium (K).

Community‐wise regression analyses revealed that only a few environmental variables were significantly associated with Shannon diversity, whereas most predictors showed no significant relationships (Figure 9; Table S5). In the QCL community, Shannon diversity was significantly related to soil texture variables, including silt (*R* ^2^ = 0.491, *p* = 0.0004) and sand (*R* ^2^ = 0.530, *p* = 0.015). In addition, pH (*R* ^2^ = 0.458, *p* = 0.041) and nitrogen (*R* ^2^ = 0.475, *p* < 0.001) were significant predictors of diversity. In the PJH community, Shannon diversity was significantly associated only with silt content (*R* ^2^ = 0.370, *p* = 0.0038). No other environmental variables showed significant relationships. In the BRI community, phosphorus exhibited a significant positive relationship with Shannon diversity (*R* ^2^ = 0.608, *p* = 0.023), whereas all other predictors were non‐significant. No significant relationships between Shannon diversity and the measured environmental variables were detected in the CDA or PSG communities. Furthermore, topographic variables (altitude and slope) did not significantly influence Shannon diversity in any of the studied communities. Shannon diversity was primarily associated with soil texture (silt and sand), soil pH, and nutrient availability (nitrogen and phosphorus), with the strongest effects observed in the QCL, PJH, and BRI communities.

## 4 Discussion

The floristic composition of the moist temperate forest reflects high taxonomic and life‐form diversity, consistent with patterns reported from temperate mountain ecosystems (Hashim et al. 2023). The dominance of herbaceous taxa and hemicryptophytic life forms reflects adaptation to the seasonal climatic conditions of montane environments (Kermavnar et al. 2022). Beyond overall floristic richness, the classification analyses revealed that vegetation was organized into five ecologically distinct communities, each characterized by diagnostic indicator species with strong fidelity to particular habitat conditions. The contrasting environmental conditions of BRI and QCL indicate that species assemblages were shaped by environmental filtering rather than random species co‐occurrence. Such community differentiation is consistent with theoretical and empirical evidence that deterministic processes govern species assembly across heterogeneous landscapes (Liu et al. 2016; Fan et al. 2017; Hou et al. 2026). The identification of indicator taxa associated with specific plant associations therefore not only provides a robust classification framework but also reflects ecological specialization along topographic and edaphic gradients. This interpretation agrees with previous studies demonstrating that indicator‐based approaches effectively capture vegetation organization and functional differentiation within forest ecosystems (Baranova et al. 2016; Campos et al. 2020).

Distinct compositional differentiation among the five plant communities reflects strong ecological segregation within the moist temperate forest. The clear clustering of plots in PCoA space indicates distinct community assemblages associated with different environmental conditions, consistent with habitat‐driven species distributions (Molozhon et al. 2023; Silva et al. 2020; Laitinen et al. 2021). The BRI community exhibited the highest Shannon diversity, Simpson diversity, and evenness, whereas QCL displayed the lowest diversity and highest dominance. BRI occurred at the highest elevations and was associated with greater silt content and relatively high nitrogen availability, factors that may support higher species coexistence. In contrast, QCL occurred at the lowest elevations and was characterized by highly sandy soils with low clay and silt contents, conditions that may limit moisture and nutrient availability. Consequently, QCL exhibited greater dominance by a limited number of taxa and lower overall diversity. Community differences were also evident in beta diversity, which was driven primarily by species turnover rather than nestedness. Complete turnover between some community pairs, particularly BRI and QCL, indicates that differences among assemblages resulted primarily from species replacement rather than progressive species loss (Peguero et al. 2022; Wu et al. 2023; Li et al. 2026). Such turnover‐dominated patterns indicate strong habitat specialization and environmental filtering across heterogeneous mountain landscapes (Xin et al. 2024; Wani et al. 2023; Saqib et al. 2024). Because beta‐diversity partitioning was based on presence–absence data, future studies incorporating abundance‐based measures such as Bray–Curtis dissimilarity may reveal additional dimensions of community differentiation.

Variation in environmental characteristics among plant communities reflects pronounced habitat heterogeneity across the moist temperate forest landscape. Significant environmental differences among communities indicate strong habitat heterogeneity across the landscape. Elevation emerged as a major gradient separating communities, with BRI occurring near 2921 m and QCL occupying substantially lower elevations around 1585 m. These differences likely influence temperature regimes, moisture availability, and soil development processes that regulate plant establishment and persistence (Wani et al. 2023; Ullah et al. 2024; Rawal et al. 2025). Soil texture exhibited similarly strong variation among communities, with QCL associated with coarse sandy substrates, whereas CDA and PJH occurred on relatively clay‐rich soils and BRI on more silt‐rich soils. Such differences are ecologically important because soil texture directly affects water retention, aeration, rooting conditions, and nutrient availability (Necer et al. 2019; Luo et al. 2024). The CCA results provide direct support for this interpretation, identifying sand, clay, and slope as the only significant environmental predictors of species composition after accounting for multicollinearity among variables. Communities were separated along gradients of sand, clay, and slope, reflecting differences in soil texture and topographic conditions. These findings suggest that physical habitat properties exert stronger control over vegetation composition than soil chemical variables at the landscape scale. Similar patterns have been reported in mountainous ecosystems, where soil texture and topography act as key filters shaping species distributions and habitat specialization (Yanyan et al. 2017; Haq et al. 2015; Berihun Tenaw et al. 2024; Pang et al. 2025).

Within communities, Shannon diversity was influenced by only a few environmental variables, suggesting that local diversity drivers differed from those shaping community composition. The strongest responses were observed in the QCL community, where diversity was significantly associated with both silt and sand content, as well as soil pH and nitrogen availability. The association of diversity with sand, silt, pH, and nitrogen in QCL suggests that local variation in soil texture and resource availability shaped diversity within this sandy community. Responses to soil texture reinforce the role of particle‐size distribution in regulating moisture availability, nutrient dynamics, and rooting conditions that collectively shape local species richness (Du and Zhang 2024; Khan and Khalid 2021). In the PJH community, diversity was associated only with silt content, highlighting the importance of soil texture and moisture retention in regulating local species richness. Soil pH also influenced diversity in QCL, suggesting that variation in nutrient availability and soil biological activity contributed to local species richness (Xin et al. 2024). Nutrient availability also influenced diversity, with nitrogen being important in QCL and phosphorus in BRI, highlighting the role of key macronutrients in shaping local diversity patterns (Matkala et al. 2020; Wang et al. 2024). In BRI, phosphorus was the only significant predictor of diversity, indicating the importance of nutrient availability in this high‐diversity community. Most other environmental variables, including clay, EC, TSS, CaCO_3_, organic matter, potassium, altitude, and slope, had little influence on within‐community diversity. These results indicate that different factors governed community composition and within‐community diversity, with the former driven by soil texture and slope and the latter by local soil and nutrient conditions. Community composition was associated with landscape‐scale heterogeneity, whereas local diversity reflected finer‐scale environmental variation. Nevertheless, the relatively low adjusted *R* ^2^ of the CCA model indicates that a substantial proportion of variation in community composition remained unexplained. This indicates that unmeasured factors, such as dispersal, disturbance, biotic interactions, and microclimatic variation, may also shape vegetation patterns.

## 5 Management Implications

The marked compositional differences among plant communities and the high species turnover observed in this study indicate that conservation efforts should focus on maintaining the full range of habitats represented across the moist temperate forest landscape. Particular attention should be given to the BRI community, which supported the highest species diversity, and to environmentally distinct communities such as QCL, which occupied low‐elevation, sandy habitats and contained unique species assemblages. Because community composition was strongly associated with soil texture and slope, management actions that alter soil conditions, including road expansion, slope modification, excessive grazing, and timber extraction, should be minimized in sensitive sites. Habitat connectivity among communities should also be maintained to preserve landscape‐level biodiversity and ecological processes. Given the increasing infrastructure development and human activities in the Lowari region, conservation planning should prioritize community‐specific management rather than uniform forest protection measures. Long‐term monitoring of vegetation and environmental change across elevation and soil gradients will be important for detecting shifts in community composition and guiding adaptive management of these moist temperate forests.

## 6 Conclusion

Plant communities in moist temperate forests were organized along elevation and soil gradients, producing distinct vegetation assemblages across relatively short spatial scales. Species turnover dominated patterns of beta diversity, reflecting strong compositional differentiation rather than progressive species loss among communities. Soil texture fractions and terrain attributes were significant correlates of community structure; however, a considerable proportion of variation remained unexplained, indicating that additional ecological processes may also contribute to community assembly in these moist temperate forests. Certain communities maintained higher evenness and structural complexity, whereas others were characterized by greater dominance. These patterns emphasize the role of fine‐scale environmental heterogeneity in governing vegetation assembly within montane forest ecosystems. Conservation and management efforts should therefore prioritize maintaining topographic and edaphic variability to conserve community‐level differentiation and ecosystem resilience under changing climatic conditions. Protection of environmentally distinct forest patches remains essential for preserving compositional integrity, sustaining biodiversity, and supporting long‐term ecological stability in moist temperate mountain landscapes.

## Author Contributions

**Mehreen Samad:** conceptualization (equal), investigation (equal), methodology (equal), writing – original draft (equal), writing – review and editing (equal). **Nadeem Ahmad:** data curation (equal), methodology (equal), supervision (equal), validation (equal), writing – original draft (equal). **Adnan Ahmad:** data curation (equal), methodology (equal), supervision (equal), writing – review and editing (equal). **Abeer Al‐Andal:** data curation (equal), validation (equal), writing – review and editing (equal). **Adam Khan:** data curation (equal), writing – review and editing (equal). **Abdul Rahman Osmani:** data curation (equal), writing – review and editing (equal). **Muhammad Waheed:** conceptualization (equal), data curation (equal), formal analysis (equal), software (equal), visualization (equal), writing – original draft (equal), writing – review and editing (equal).

## Funding

This work was supported by King Khalid University, RGP2/75/46.

## Conflicts of Interest

The authors declare no conflicts of interest.

## Supporting information


**Table S1:** List of plant species with family, species code, Growth forms, and life history traits.
**Table S2:** Indicator species of the five plant communities identified through hierarchical cluster analysis and Indicator Value (IndVal) analysis.
**Table S3:** Pairwise comparisons of alpha diversity indices among plant communities showing estimated differences, standard errors (SE), *t*‐ratios, and adjusted *p‐*values.
**Table S4:** Pairwise comparisons of environmental and soil variables among plant communities showing estimated differences, standard errors (SE), *t*‐ratios, and adjusted *p*‐values.
**Table S5:** Community‐wise model statistics describing relationships between Shannon diversity and environmental variables, including sample size (*n*), coefficient of determination (*R* ^2^), adjusted *R* ^2^, and residual standard deviation (*σ*).
**Table S6:** Independent contributions of environmental predictors from hierarchical partitioning.
**Table S7:** Beta‐deviation null‐model test for βSIM turnover.

**Figure S1:** Pearson correlation matrix among environmental variables used in the canonical correspondence analysis (CCA). Colors indicate the direction and strength of pairwise correlations, with blue representing positive correlations and red representing negative correlations. Values within cells represent Pearson correlation coefficients (*r*).
**Figure S2:** Beta‐deviation null‐model test for species turnover among plant communities. Gray bars show the null distribution of between‐minus‐within βSIM values generated by randomizing the occurrence matrix while preserving site richness and species occupancy. The red vertical line represents the observed value.
**Figure S3:** Hierarchical partitioning of independent contributions for the selected CCA predictors. Bars show the independent adjusted *R* ^2^ contribution of sand, clay, and slope to community composition.
**Figure S4:** Hierarchical partitioning after adding altitude to the selected CCA predictors. Bars show the independent adjusted *R* ^2^ contribution of sand, clay, slope, and altitude. Altitude showed no significant independent contribution.
**Figure S5:** Variance partitioning of Hellinger‐transformed community composition between edaphic and topographic predictor sets. Values represent adjusted *R* ^2^ fractions for pure edaphic, pure topographic, shared, and residual components.

## Data Availability

Data used in this study is available in the main text and as Supporting Information of this paper.
