# Supplementary material for: Species Turnover and Environmental Filtering Structure Plant Communities in Moist Temperate Forests
Source: Ecol Evol. 2026 Jul 29;16(8):e74054. doi: 10.1002/ece3.74054 (PMC13420373; doi:10.1002/ece3.74054)
Supplement: Supplementary file 1 — Table S1: List of plant species with family, species code, Growth forms, and life history traits. Table S2: Indicator species of the five plant communities identified through hierarchical cluster analysis and Indicator Value (IndVal) analysis. Table S3: Pairwise comparisons of alpha diversity indices among plant communities showing estimated differences, standard errors (SE), t‐ratios, and adjusted p‐values. Table S4: Pairwise comparisons of environmental and soil variables among plant communities showing estimated differences, standard errors (SE), t‐ratios, and adjusted p‐values. Table S5: Community‐wise model statistics describing relationships between Shannon diversity and environmental variables, including sample size (n), coefficient of determination (R 2), adjusted R 2, and residual standard deviation (σ). Table S6: Independent contributions of environmental predictors from hierarchical partitioning. Table S7: Beta‐deviation null‐model test for βSIM turnover. [file ECE3-16-e74054-s001.docx]

**Additional file Table S1.** List of plant species with family, species code, Growth forms, and life history traits.

| Voucher No. | Division | Species | Species | Habit | Family | Life Form | Leaf size |
| --- | --- | --- | --- | --- | --- | --- | --- |
| M. Samad Bot. 8 (PUP) | Gymnosperm | *Cedrus deodar* Roxb. Ex D. Don | Ced-deo | Tree | Pinaceae | Microphanerophytes | Nanophyll |
| M. Samad Bot. 4 (PUP) | Gymnosperm | *Pinus wallichiana* AB Jackson | Pin-wal | Tree | Pinaceae | Microphanerophytes | Nanophyll |
| M. Samad Bot. 9 (PUP) | Gymnosperm | *Abies pindrow* (Royle) | Abi-pin | Tree | Pinaceae | Microphanerophytes | Nanophyll |
| M. Samad Bot. 1 (PUP) | Dicotyledon | *Quercus baloot* Griff | Que-bal | Shrub | Fagaceae | Mesophanerophytes | Mesophyll |
| M. Samad Bot. 2 (PUP) | Gymnosperm | *Picea smithiana* (Wall) | Pic-smi | Tree | Pinaceae | Megaphanerophytes | Nanophyll |
| M. Samad Bot. 38 (PUP) | Dicotyledon | *Populus ciliata* Wall.ex Royle | Pop-cil | Tree | Salicaceae | Microphanerophytes | Mesophyll |
| M. Samad Bot. 11 (PUP) | Dicotyledon | *Juglans regia* L. | Jug-reg | Tree | Juglandaceae | Mesophanerophytes | Mesophyll |
| M. Samad Bot. 7 (PUP) | Dicotyledon | *Melaia azedarach* Linn. | Mel-aze | Tree | Meliaceae | Mesophanerophytes | Microphyll |
| M. Samad Bot. 12 (PUP) | Dicotyledon | *Diospyros lotus* L. | Dio-lot | Shrub | Ebencaceae | Mesophanerophytes | Microphyll |
| M. Samad Bot. 5 (PUP) | Gymnosperm | *Juniperus indica* Bertol | Jun-ind | Shrub | Cupressaceae | Microphanerophytes | Leptophyll |
| M. Samad Bot. 3 (PUP) | Monocotyledon | *Astragalus pyrrhotrichus* Boiss. | Ast-pyr | Shrub | Fabaceae | Chamaephytes | Microphyll |
| M. Samad Bot. 10 (PUP) | Dicotyledon | *Cotoneaster nummularius* Fisch & C. A. Meyer | Cot-num | Shrub | Rosaceae | Nanophanerophytes | Microphyll |
| M. Samad Bot. 17 (PUP) | Dicotyledon | *Indigofera articulata* Wall. Ex Brand | Ind-art | Shrub | Fabaceae | Chamaephytes | Microphyll |
| M. Samad Bot. 14 (PUP) | Dicotyledon | *Verbascum thapsus* L. | Ver-tha | Herb | Scrophulariaceae | Hemicryptophytes | Mesophyll |
| M. Samad Bot. 25 (PUP) | Monocotyledon | *Cynodon dactylon* (L.) Pers | Cyn-dac | Herb | Poaceae | Hemicryptophytes | Nanophyll |
| M. Samad Bot. 15 (PUP) | Dicotyledon | *Artemisia vulagaris* L. | Art-vul | Herb | Asteraceae | Hemicryptophytes | Mesophyll |
| M. Samad Bot. 24 (PUP) | Dicotyledon | *Trifolium repens* L. | Tri-rep | Herb | Fabaceae | Chamaephytes | Microphyll |
| M. Samad Bot. 13 (PUP) | Dicotyledon | *Lamium album* L. | Lam-alb | Herb | Lamiaceae | Hemicryptophytes | Microphyll |
| M. Samad Bot. 26 (PUP) | Dicotyledon | *Nepeta raphanorhiza* Benth. | Nep-rap | Herb | Lamiaceae | Hemicryptophytes | Microphyll |
| M. Samad Bot. 16 (PUP) | Dicotyledon | *Carduus collinus* Waldst and Kit | Car-col | Herb | Asteraceae | Hemicryptophytes | Macrophyll |
| M. Samad Bot. 31 (PUP) | Dicotyledon | *Delphinium nordhagenni* wedelbo | Del-nor | Herb | Ranunculaceae | Hemicryptophytes | Mesophyll |
| M. Samad Bot. 28 (PUP) | Dicotyledon | *Herniaria hirasuta* Linn. | Her-hir | Herb | Caryophyllaceae | Therophytes | Leptophyll |
| M. Samad Bot. 34 (PUP) | Dicotyledon | *Vicia tenuifolia* Roth. | Vic-ten | Herb | Fabaceae | Chamaephytes | Nanophyll |
| M. Samad Bot. 36 (PUP) | Dicotyledon | *Aconitum lycoctonum* L. | Aco-lyc | Herb | Ranunculaceae | Hemicryptophytes | Mesophyll |
| M. Samad Bot. 27 (PUP) | Monocotyledon | *Stipa orientalis* L. | Sti-ori | Herb | Poaceae | Hemicryptophytes | Nanophyll |
| M. Samad Bot. 32 (PUP) | Dicotyledon | *Cynoglossum amabile* Stapf and J.R.Drumm | Cyn-ama | Herb | Boraginaceae | Hemicryptophytes | Mesophyll |
| M. Samad Bot. 69 (PUP) | Dicotyledon | *Anthemis cotula* L. | Ant-cot | Herb | Asteraceae | Hemicryptophytes | Microphyll |
| M. Samad Bot. 37 (PUP) | Monocotyledon | *Schkuhria pinnata* (Lam.) Kuntze ex Thell. | Sch-pin | Herb | Asteraceae | Therophytes | Nanophyll |
| M. Samad Bot. 20 (PUP) | Dicotyledon | *Cirsium argyracanthum* Wall ex. | cir-arg | Herb | Asteraceae | Hemicryptophytes | Macrophyll |
| M. Samad Bot. 26 (PUP) | Dicotyledon | *Chenopodium vulgare* L. | Chn-vul. | Herb | Lamiaceae | Hemicryptophytes | Microphyll |
| M. Samad Bot. 19 (PUP) | Dicotyledon | *Rumex dentatus* L. | Rum-den | Herb | Polygonaceae | Therophytes | Mesophyll |
| M. Samad Bot. 22 (PUP) | Dicotyledon | *Rumex hastatus* S.D.Don | Rum-has | Shrub | Polygonaceae | Therophytes | Microphyll |
| M. Samad Bot. 23 (PUP) | Dicotyledon | *Lactuca virosa* L. | Lac-vir | Herb | Asteraceae | Hemicryptophytes | Macrophyll |
| M. Samad Bot. 21 (PUP) | Monocotyledon | *Asperula lilaciflora* Boiss | Asp-lil | Shrub | Rubiaceae | Chamaephytes | Microphyll |
| M. Samad Bot. 18 (PUP) | Monocotyledon | *Carex diluta* M. Bieb | Car-dil | Herb | Cyperaceae | Hemicryptophytes | Nanophyll |
| M. Samad Bot. 29 (PUP) | Dicotyledon | *Fragaria vesca* L. | Fra-ves | Shrub | Rosaceae | Hemicryptophytes | Microphyll |
| M. Samad Bot. 70 (PUP) | Monocotyledon | *Cymbopogon jwarancusa* Jones | Cym-jwa | Herb | Poaceae | Hemicryptophytes | Nanophyll |
| M. Samad Bot. 68 (PUP) | Dicotyledon | Avena fatua L. | Ave-fat | Herb | Poaceae | Therophytes | Nanophyll |
| M. Samad Bot. 30 (PUP) | Monocotyledon | *Aconitum chasmanthum* Stapf ex. Holmes | Aco-cha | Shrub | Ranunculaceae | Hemicryptophytes | Mesophyll |
| M. Samad Bot. 33 (PUP) | Monocotyledon | *Acorus calamus* L. | Aco-cal | Shrub | Acoraceae | Hemicryptophytes | Microphyll |
| M. Samad Bot. 67 (PUP) | Dicotyledon | *Ajuga bracteosa* Wall. Ex Benth | Aju-bra | Herb | Limiaceae | Hemicryptophytes | Microphyll |
| M. Samad Bot. 41 (PUP) | Dicotyledon | *Amaranthus spinosus* L. | Ama-spi | Herb | Amaranthaceae | Therophytes | Microphyll |
| M. Samad Bot. 44 (PUP) | Dicotyledon | *Berberis lycium* L. | Ber-lyc | Shrub | Solanaceae | Nanophanerophytes | Microphyll |
| M. Samad Bot. 39 (PUP) | Dicotyledon | *Trigonella foenum-graecum* L. | Tri-foe | Herb | Fabaceae | Chamaephytes | Microphyll |
| M. Samad Bot. 64 (PUP) | Dicotyledon | *Hedera nepalensis* K. Koch Hort Dendrol | Hed-nep | Herb | Araliaceae | Nanophanerophytes | Mesophyll |
| M. Samad Bot. 66 (PUP) | Dicotyledon | *Justicia adhatoda* L. | Jus-adh | Shrub | Acanthaceae | Nanophanerophytes | Mesophyll |
| M. Samad Bot. 65 (PUP) | Dicotyledon | *Mentha longifolia* L. | Men-lon | Herb | Lamiaceae | Hemicryptophytes | Mesophyll |
| M. Samad Bot. 43 (PUP) | Monocotyledon | *Origanum vulgare* L. | Ori-vul | Herb | Lamiaceae | Hemicryptophytes | Microphyll |
| M. Samad Bot. 63 (PUP) | Dicotyledon | *Oxalis corniculata* L. | Oxa-cor | Herb | Oxalidiaceae | Geophytes | Nanophyll |
| M. Samad Bot. 40 (PUP) | Dicotyledon | *Paeonia officinalis* (L) | Pae-off | Herb | Paeonicaceae | Hemicryptophytes | Macrophyll |
| M. Samad Bot. 35 (PUP) | Dicotyledon | *Ricinus communis* L. | Ric-com | Shrub | Euphorbiaceae | Nanophanerophytes | Macrophyll |
| M. Samad Bot. 42 (PUP) | Gymnosperm | *Pinus roxburghii* Roxb. | Pin-rox | Tree | Pinaceae | Mesophanerophytes | Nanophyll |
| M. Samad Bot. 45 (PUP) | Dicotyledon | *Anaphalis chitralensis* Qaiser and Abid | Ana-chi | Shrub | Asteraceae | Chamaephytes | Microphyll |
| M. Samad Bot. 47 (PUP) | Dicotyledon | *Astragalus lowarensis* Ali | Ast-low | Herb | Fabaceae | Chamaephytes | Microphyll |
| M. Samad Bot. 51 (PUP) | Dicotyledon | *Delphinium kohatense* (BrohD Munz) | Del-koh | Herb | Ranunculaceae | Hemicryptophytes | Mesophyll |
| M. Samad Bot. 48 (PUP) | Dicotyledon | *Plocama asperuliformis* (Lincz) M. Backlund and Thulin | Plo-asp | Shrub | Rubiaceae | Chamaephytes | Nanophyll |
| M. Samad Bot. 52 (PUP) | Dicotyledon | *Galium ghilanicum* Stapf | Gal-ghi | Herb | Rubiaceae | Therophytes | Nanophyll |
| M. Samad Bot. 46 (PUP) | Dicotyledon | *Oxytropis gloriasa* Ali | Oxy- glo | Herb | Fabaceae | Therophytes | Microphyll |
| M. Samad Bot. 50 (PUP) | Dicotyledon | *Polygonum cognatum* (Rech. F. and Schiman-Czeika) Qaiser | Pol-cog | Herb | Polygonaceae | Therophytes | Microphyll |
| M. Samad Bot. 49 (PUP) | Dicotyledon | *Silene logisepala* Nasir | Sil-log | Shrub | Caryophyllaceae | Hemicryptophytes | Microphyll |
| M. Samad Bot. 55 (PUP) | Dicotyledon | *Psychrogeton chitralicus* Gierson | Psy-chi | Herb | Asteraceae | Hemicryptophytes | Microphyll |
| M. Samad Bot. 54 (PUP) | Dicotyledon | *Impatiens lemannii* Hook. F and Thomson | Imp-lem | Herb | Balsaminaceae | Hemicryptophytes | Microphyll |
| M. Samad Bot. 57 (PUP) | Dicotyledon | *Strigosella toppinii* (O.E. Schulz) Botsch | Str-top | Herb | Brassicaceae | Therophytes | Microphyll |
| M. Samad Bot. 59 (PUP) | Dicotyledon | *Calotropis procera* (Ait.) Ait. | Cal-pro | Shrub | Apocynaceae | Nanophanerophytes | Mesophyll |
| M. Samad Bot. 53 (PUP) | Dicotyledon | *Nerium indicum* Mill. | Ner-ind | Shrub | Apocynaceae | Nanophanerophytes | Mesophyll |
| M. Samad Bot. 62 (PUP) | Dicotyledon | *Daphne mucronata* Royle | Dap-muc | Shrub | Thymelaeaceae | Nanophanerophytes | Microphyll |
| M. Samad Bot. 58 (PUP) | Dicotyledon | *Descurainia sophia* (L.) Webb and Berth | Des-sop | Herb | Brassicaceae | Therophytes | Nanophyll |
| M. Samad Bot. 56 (PUP) | Dicotyledon | *Elaeagnus angustifolia* L. | Ela-ang | Tree | Eleagnaceae | Mesophanerophytes | Microphyll |
| M. Samad Bot. 61 (PUP) | Monocotyledon | *Ephedra intermedia* Schrenk and Meyer | Eph-int | Shrub | Ephedraceae | Nanophanerophytes | Nanophyll |
| M. Samad Bot. 60 (PUP) | Monocotyledon | *Eremurus stenophyllus* (Boiss and Buhse) | Ere-ste | Herb | Asphodelaceae | Hemicryptophytes | Nanophyll |

**Additional File Table S2.** Indicator species of the five plant communities identified through hierarchical cluster analysis and Indicator Value (IndVal) analysis.

| Species | BRI | | CDA | | PSG | | QCL | | PJH | |
| --- | --- | --- | --- | --- | --- | --- | --- | --- | --- | --- |
|  | IndVal | *P* value | IndVal | *P* value | IndVal | *P* value | IndVal | *P* value | IndVal | *P* value |
| *Astragalus pyrrhotrichus* | 0 | 0 | 26.67 | 0.6 | 0 | 0 | 0 | 0 | 0 | 0 |
| *Abies pindrow* | 24.24 | 0.47 | 0 | 0 | 0 | 0 | 0 | 0 | 0 | 0 |
| *Aconitum chasmanthum* | 0 | 0 | 0 | 0 | 0 | 0 | 18.18 | 0.43 | 0 | 0 |
| *Ajuga bracteosa* | 0 | 0 | 6.67 | 0.286 | 0 | 0 | 0 | 0 | 0 | 0 |
| *Amaranthus spinosus* | 0 | 0 | 0 | 0 | 0 | 0 | 0 | 0 | 1 | 0.27 |
| *Artemisia vulagaris* | 0 | 0 | 0 | 0 | 0 | 0 | 45.45 | 0.1 | 0 | 0 |
| *Avena fatua* | 0 | 0 | 0 | 0 | 0 | 0 | 9.9 | 0.217 | 0 | 0 |
| *Berberis lycium* | 1 | 0.1 | 0 | 0 | 0 | 0 | 0 | 0 | 0 | 0 |
| *Calotropis procera* | 0 | 0 | 0 | 0 | 12.5 | 0.157 | 0 | 0 | 0 | 0 |
| *Carex diluta* | 0 | 0 | 0 | 0 | 0 | 0 | 81.82 | 0.1 | 0 | 0 |
| *Cedrus deodara* | 0 | 0 | 53.33 | 0.1 | 0 | 0 | 0 | 0 | 4.46 | 0.955 |
| *Cynodon dactylon* | 9.26 | 0.66 | 5.93 | 0.219 | 0 | 0 | 0 | 0 | 0 | 0 |
| *Daphne mucronata* | 0 | 0 | 0 | 0 | 25 | 0.17 | 0 | 0 | 0 | 0 |
| *Delphinium kohatense* | 0 | 0 | 0 | 0 | 25 | 0.17 | 0 | 0 | 0 | 0 |
| *Delphinium nordhagenii* | 0 | 0 | 34.52 | 0.2 | 0 | 0 | 0 | 0 | 0 | 0 |
| *Ephedra intermedia* | 0 | 0 | 0 | 0 | 25 | 0.19 | 0 | 0 | 0 | 0 |
| *Eremurus stenophyllus* | 0 | 0 | 0 | 0 | 25 | 0.18 | 0 | 0 | 0 | 0 |
| *Fragaria vesca* | 14.81 | 0.49 | 11.11 | 0.13 | 0 | 0 | 0 | 0 | 0 | 0 |
| *Galium ghilanicum* | 0 | 0 | 0 | 0 | 3.18 | 0.6 | 1.77 | 0.592 | 0 | 0 |
| *Hedera nepalensis* | 0 | 0 | 0 | 0 | 0 | 0 | 0 | 0 | 67.99 | 0.1 |
| *Indigofera articulata* | 36.56 | 0.8 | 0 | 0 | 0 | 0 | 0 | 0 | 0 | 0 |
| *Juniperus indica* | 0 | 0 | 6.67 | 0.29 | 0 | 0 | 0 | 0 | 7 | 0.1 |
| *Lactuca virosa* | 0 | 0 | 0 | 0 | 0 | 0 | 63.64 | 0.1 | 0 | 0 |
| *Lamium album* | 26.73 | 0.8 | 0 | 0 | 0 | 0 | 0 | 0 | 0 | 0 |
| *Nepeta raphanorhiza* | 0 | 0 | 6.67 | 0.29 | 0 | 0 | 0 | 0 | 0 | 0 |
| *Nerium indicum* | 0 | 0 | 0 | 0 | 25 | 0.27 | 0 | 0 | 0 | 0 |
| *Oxalis corniculata* | 0 | 0 | 0 | 0 | 0 | 0 | 0 | 0 | 3 | 0.8 |
| *Paeonia officinalis* | 0 | 0 | 0 | 0 | 0 | 0 | 0 | 0 | 2 | 0.28 |
| *Picea smithiana* | 0 | 0 | 0 | 0 | 62.5 | 0.1 | 0 | 0 | 0 | 0 |
| *Pinus wallichiana* | 0 | 0 | 0 | 0 | 0 | 0 | 2.81 | 0.866 | 9 | 0.1 |
| *Quercus baloot* | 0 | 0 | 0 | 0 | 0 | 0 | 81.82 | 0.1 | 0 | 0 |
| *Ricinus communis* | 0 | 0 | 0 | 0 | 0 | 0 | 0 | 0 | 5 | 0.1 |
| *Rumex dentatus* | 66.67 | 0.1 | 0 | 0 | 0 | 0 | 0 | 0 | 0 | 0 |
| *Rumex hastatus* | 0 | 0 | 6.67 | 0.29 | 0 | 0 | 0 | 0 | 0 | 0 |
| *Schkuhria pinnata* | 0 | 0 | 13.33 | 0.96 | 0 | 0 | 0 | 0 | 0 | 0 |
| *Silene logisepala* | 0 | 0 | 0 | 0 | 37.5 | 0.4 | 0 | 0 | 0 | 0 |
| *Trifolium repens* | 16.67 | 0.17 | 0 | 0 | 0 | 0 | 0 | 0 | 0 | 0 |
| *Verbascum thapsus* | 0 | 0 | 26.67 | 0.6 | 0 | 0 | 0 | 0 | 0 | 0 |
| *Vicia tenuifolia* | 0 | 0 | 6.67 | 0.29 | 0 | 0 | 0 | 0 | 0 | 0 |

**Additional file; Table S3.** Pairwise comparisons of alpha diversity indices among plant communities showing estimated differences, standard errors (SE), t-ratios, and adjusted *p-*values.

| Indices | contrast | estimate | SE | *t* ratio | *p* value |
| --- | --- | --- | --- | --- | --- |
| Shannon | BRI - CDA | 0.17151 | 0.17185852 | 0.99797204 | 0.85487766 |
|  | BRI - QCL | 0.59392091 | 0.16790722 | 3.53719689 | 0.0080469 |
|  | BRI - PJH | 0.26095727 | 0.16790722 | 1.55417538 | 0.53376249 |
|  | BRI - PSG | 0.2725925 | 0.18228349 | 1.49543165 | 0.57073516 |
|  | CDA - QCL | 0.42241091 | 0.16790722 | 2.51573994 | 0.10508831 |
|  | CDA - PJH | 0.08944727 | 0.16790722 | 0.53271843 | 0.98346718 |
|  | CDA - PSG | 0.1010825 | 0.18228349 | 0.55453459 | 0.98081356 |
|  | QCL - PJH | -0.33296364 | 0.16386067 | -2.03199238 | 0.26777073 |
|  | QCL - PSG | -0.32132841 | 0.17856303 | -1.79952376 | 0.38652748 |
|  | PJH - PSG | 0.01163523 | 0.17856303 | 0.06516034 | 0.99999574 |
| Simpson | BRI - CDA | 0.09597 | 0.09315852 | 1.03017955 | 0.84006002 |
|  | BRI - QCL | 0.35042091 | 0.09101666 | 3.8500745 | 0.0032531 |
|  | BRI - PJH | 0.14660636 | 0.09101666 | 1.61076411 | 0.49850964 |
|  | BRI - PSG | 0.16117 | 0.09880953 | 1.63111797 | 0.48596639 |
|  | CDA - QCL | 0.25445091 | 0.09101666 | 2.79565212 | 0.05577604 |
|  | CDA - PJH | 0.05063636 | 0.09101666 | 0.55634172 | 0.98058143 |
|  | CDA - PSG | 0.0652 | 0.09880953 | 0.65985538 | 0.96386595 |
|  | QCL - PJH | -0.20381455 | 0.08882316 | -2.29461034 | 0.16555083 |
|  | QCL - PSG | -0.18925091 | 0.0967928 | -1.95521687 | 0.30420249 |
|  | PJH - PSG | 0.01456364 | 0.0967928 | 0.15046199 | 0.99988011 |
| Dominance | BRI - CDA | -0.095968 | 0.09315854 | -1.03015785 | 0.84007024 |
|  | BRI - QCL | -0.35042255 | 0.09101668 | -3.85009162 | 0.00325293 |
|  | BRI - PJH | -0.14660436 | 0.09101668 | -1.61074177 | 0.49852345 |
|  | BRI - PSG | -0.161168 | 0.09880955 | -1.63109737 | 0.48597905 |
|  | CDA - QCL | -0.25445455 | 0.09101668 | -2.79569144 | 0.05577084 |
|  | CDA - PJH | -0.05063636 | 0.09101668 | -0.5563416 | 0.98058145 |
|  | CDA - PSG | -0.0652 | 0.09880955 | -0.65985523 | 0.96386598 |
|  | QCL - PJH | 0.20381818 | 0.08882318 | 2.29465076 | 0.16553774 |
|  | QCL - PSG | 0.18925455 | 0.09679282 | 1.955254 | 0.30418417 |
|  | PJH - PSG | -0.01456364 | 0.09679282 | -0.15046195 | 0.99988011 |
| Evenness | BRI - CDA | 0.07241 | 0.08836711 | 0.8194225 | 0.92326931 |
|  | BRI - QCL | 0.25222818 | 0.08633541 | 2.92149157 | 0.04115612 |
|  | BRI - PJH | 0.20894636 | 0.08633541 | 2.42016985 | 0.12857141 |
|  | BRI - PSG | 0.148135 | 0.09372748 | 1.58048637 | 0.51731124 |
|  | CDA - QCL | 0.17981818 | 0.08633541 | 2.08278591 | 0.24528365 |
|  | CDA - PJH | 0.13653636 | 0.08633541 | 1.58146418 | 0.51670169 |
|  | CDA - PSG | 0.075725 | 0.09372748 | 0.80792743 | 0.92683877 |
|  | QCL - PJH | -0.04328182 | 0.08425474 | -0.5137019 | 0.98556326 |
|  | QCL - PSG | -0.10409318 | 0.09181447 | -1.13373395 | 0.78788618 |
|  | PJH - PSG | -0.06081136 | 0.09181447 | -0.66232876 | 0.96337932 |

**Additional file; Table S4.** Pairwise comparisons of environmental and soil variables among plant communities showing estimated differences, standard errors (SE), t-ratios, and adjusted p-values.

| Variable | contrast | estimate | SE | t.ratio | p.value |
| --- | --- | --- | --- | --- | --- |
| Altitude | BRI - CDA | 493.2389 | 71.50869 | 6.897607 | 1.42E-07 |
|  | BRI - QCL | 1336.132 | 69.8646 | 19.1246 | 2.4E-13 |
|  | BRI - PJH | 725.0933 | 69.8646 | 10.37855 | 1.87E-12 |
|  | BRI - PSG | 457.7944 | 75.84642 | 6.035807 | 2.68E-06 |
|  | CDA - QCL | 842.8935 | 69.8646 | 12.06467 | 2.62E-13 |
|  | CDA - PJH | 231.8544 | 69.8646 | 3.318625 | 0.014728 |
|  | CDA - PSG | -35.4445 | 75.84642 | -0.46732 | 0.989887 |
|  | QCL - PJH | -611.039 | 68.18086 | -8.96203 | 1.43E-10 |
|  | QCL - PSG | -878.338 | 74.29838 | -11.8218 | 2.76E-13 |
|  | PJH - PSG | -267.299 | 74.29838 | -3.59764 | 0.006779 |
| Clay | BRI - CDA | -3.3 | 0.489276 | -6.74466 | 2.39E-07 |
|  | BRI - QCL | 0.647273 | 0.478026 | 1.354052 | 0.659454 |
|  | BRI - PJH | -2.68909 | 0.478026 | -5.6254 | 1.08E-05 |
|  | BRI - PSG | 0.17 | 0.518955 | 0.327581 | 0.997419 |
|  | CDA - QCL | 3.947273 | 0.478026 | 8.257435 | 1.44E-09 |
|  | CDA - PJH | 0.610909 | 0.478026 | 1.277982 | 0.705801 |
|  | CDA - PSG | 3.47 | 0.518955 | 6.686511 | 2.92E-07 |
|  | QCL - PJH | -3.33636 | 0.466506 | -7.15181 | 5.97E-08 |
|  | QCL - PSG | -0.47727 | 0.508363 | -0.93884 | 0.880172 |
|  | PJH - PSG | 2.859091 | 0.508363 | 5.624111 | 1.08E-05 |
| Silt | BRI - CDA | 11.26 | 4.3132 | 2.610591 | 0.085377 |
|  | BRI - QCL | 19.16818 | 4.214032 | 4.548656 | 0.000376 |
|  | BRI - PJH | 10.05 | 4.214032 | 2.384889 | 0.138233 |
|  | BRI - PSG | 11.05 | 4.574839 | 2.415386 | 0.129849 |
|  | CDA - QCL | 7.908182 | 4.214032 | 1.876631 | 0.344416 |
|  | CDA - PJH | -1.21 | 4.214032 | -0.28714 | 0.998457 |
|  | CDA - PSG | -0.21 | 4.574839 | -0.0459 | 0.999999 |
|  | QCL - PJH | -9.11818 | 4.112474 | -2.2172 | 0.192085 |
|  | QCL - PSG | -8.11818 | 4.481465 | -1.8115 | 0.379825 |
|  | PJH - PSG | 1 | 4.481465 | 0.223141 | 0.999428 |
| Sand | BRI - CDA | -9.22 | 4.506354 | -2.046 | 0.261439 |
|  | BRI - QCL | -21.0755 | 4.402746 | -4.78689 | 0.000175 |
|  | BRI - PJH | -8.43909 | 4.402746 | -1.91678 | 0.323514 |
|  | BRI - PSG | -12.48 | 4.77971 | -2.61104 | 0.085292 |
|  | CDA - QCL | -11.8555 | 4.402746 | -2.69274 | 0.070905 |
|  | CDA - PJH | 0.780909 | 4.402746 | 0.177369 | 0.99977 |
|  | CDA - PSG | -3.26 | 4.77971 | -0.68205 | 0.959345 |
|  | QCL - PJH | 12.63636 | 4.29664 | 2.940987 | 0.039224 |
|  | QCL - PSG | 8.595455 | 4.682155 | 1.83579 | 0.366411 |
|  | PJH - PSG | -4.04091 | 4.682155 | -0.86304 | 0.908794 |
| pH | BRI - CDA | 0.5136 | 0.083385 | 6.159386 | 1.76E-06 |
|  | BRI - QCL | 0.240909 | 0.081468 | 2.957109 | 0.037687 |
|  | BRI - PJH | 0.222727 | 0.081468 | 2.733931 | 0.064472 |
|  | BRI - PSG | 0.175 | 0.088443 | 1.978674 | 0.292765 |
|  | CDA - QCL | -0.27269 | 0.081468 | -3.34722 | 0.013628 |
|  | CDA - PJH | -0.29087 | 0.081468 | -3.5704 | 0.007325 |
|  | CDA - PSG | -0.3386 | 0.088443 | -3.82845 | 0.003468 |
|  | QCL - PJH | -0.01818 | 0.079504 | -0.22869 | 0.99937 |
|  | QCL - PSG | -0.06591 | 0.086638 | -0.76074 | 0.940425 |
|  | PJH - PSG | -0.04773 | 0.086638 | -0.55088 | 0.981277 |
| EC | BRI - CDA | -0.5161 | 0.113694 | -4.53939 | 0.000388 |
|  | BRI - QCL | -0.3471 | 0.11108 | -3.12478 | 0.024621 |
|  | BRI - PJH | -0.42528 | 0.11108 | -3.82862 | 0.003466 |
|  | BRI - PSG | -0.36335 | 0.12059 | -3.01309 | 0.03276 |
|  | CDA - QCL | 0.169 | 0.11108 | 1.521429 | 0.554344 |
|  | CDA - PJH | 0.090818 | 0.11108 | 0.817594 | 0.923844 |
|  | CDA - PSG | 0.15275 | 0.12059 | 1.266684 | 0.71254 |
|  | QCL - PJH | -0.07818 | 0.108403 | -0.72122 | 0.950502 |
|  | QCL - PSG | -0.01625 | 0.118129 | -0.13756 | 0.999916 |
|  | PJH - PSG | 0.061932 | 0.118129 | 0.524272 | 0.984423 |
| Tss | BRI - CDA | 0.0039 | 0.002848 | 1.369146 | 0.650094 |
|  | BRI - QCL | 0.009627 | 0.002783 | 3.459315 | 0.010009 |
|  | BRI - PJH | 0.004264 | 0.002783 | 1.532029 | 0.547671 |
|  | BRI - PSG | 0.0059 | 0.003021 | 1.952814 | 0.305389 |
|  | CDA - QCL | 0.005727 | 0.002783 | 2.05795 | 0.256116 |
|  | CDA - PJH | 0.000364 | 0.002783 | 0.130663 | 0.999932 |
|  | CDA - PSG | 0.002 | 0.003021 | 0.661971 | 0.96345 |
|  | QCL - PJH | -0.00536 | 0.002716 | -1.97488 | 0.294596 |
|  | QCL - PSG | -0.00373 | 0.00296 | -1.25938 | 0.716874 |
|  | PJH - PSG | 0.001636 | 0.00296 | 0.552897 | 0.981022 |
| CaCO3 | BRI - CDA | 0.169 | 0.244332 | 0.691681 | 0.957274 |
|  | BRI - QCL | 0.255364 | 0.238715 | 1.069745 | 0.820909 |
|  | BRI - PJH | 0.350818 | 0.238715 | 1.469614 | 0.587031 |
|  | BRI - PSG | 0.52275 | 0.259153 | 2.017145 | 0.274589 |
|  | CDA - QCL | 0.086364 | 0.238715 | 0.361786 | 0.996205 |
|  | CDA - PJH | 0.181818 | 0.238715 | 0.761655 | 0.940178 |
|  | CDA - PSG | 0.35375 | 0.259153 | 1.365022 | 0.652656 |
|  | QCL - PJH | 0.095455 | 0.232962 | 0.409744 | 0.993871 |
|  | QCL - PSG | 0.267386 | 0.253864 | 1.053266 | 0.829009 |
|  | PJH - PSG | 0.171932 | 0.253864 | 0.67726 | 0.96035 |
| OM | BRI - CDA | -0.953 | 0.210298 | -4.53167 | 0.000397 |
|  | BRI - QCL | -0.23609 | 0.205463 | -1.14907 | 0.779625 |
|  | BRI - PJH | -0.60973 | 0.205463 | -2.96758 | 0.036718 |
|  | BRI - PSG | -0.60825 | 0.223054 | -2.72691 | 0.065531 |
|  | CDA - QCL | 0.716909 | 0.205463 | 3.489244 | 0.009208 |
|  | CDA - PJH | 0.343273 | 0.205463 | 1.670731 | 0.461821 |
|  | CDA - PSG | 0.34475 | 0.223054 | 1.545588 | 0.53915 |
|  | QCL - PJH | -0.37364 | 0.200511 | -1.86342 | 0.351451 |
|  | QCL - PSG | -0.37216 | 0.218502 | -1.70323 | 0.442318 |
|  | PJH - PSG | 0.001477 | 0.218502 | 0.006761 | 1 |
| N | BRI - CDA | 0.0567 | 0.024193 | 2.343681 | 0.15023 |
|  | BRI - QCL | 0.070755 | 0.023636 | 2.993446 | 0.034419 |
|  | BRI - PJH | 0.067936 | 0.023636 | 2.874216 | 0.046196 |
|  | BRI - PSG | 0.080675 | 0.02566 | 3.143968 | 0.023423 |
|  | CDA - QCL | 0.014055 | 0.023636 | 0.594612 | 0.975203 |
|  | CDA - PJH | 0.011236 | 0.023636 | 0.475382 | 0.989212 |
|  | CDA - PSG | 0.023975 | 0.02566 | 0.934325 | 0.882 |
|  | QCL - PJH | -0.00282 | 0.023067 | -0.12217 | 0.999948 |
|  | QCL - PSG | 0.00992 | 0.025137 | 0.394663 | 0.994693 |
|  | PJH - PSG | 0.012739 | 0.025137 | 0.506778 | 0.986278 |
| P | BRI - CDA | -1.89 | 0.453708 | -4.16568 | 0.001253 |
|  | BRI - QCL | -1.58 | 0.443276 | -3.56437 | 0.007452 |
|  | BRI - PJH | -0.96182 | 0.443276 | -2.1698 | 0.209803 |
|  | BRI - PSG | -1.355 | 0.481229 | -2.8157 | 0.053179 |
|  | CDA - QCL | 0.31 | 0.443276 | 0.699338 | 0.95558 |
|  | CDA - PJH | 0.928182 | 0.443276 | 2.093914 | 0.240532 |
|  | CDA - PSG | 0.535 | 0.481229 | 1.111736 | 0.799509 |
|  | QCL - PJH | 0.618182 | 0.432593 | 1.429014 | 0.612625 |
|  | QCL - PSG | 0.225 | 0.471407 | 0.477294 | 0.989047 |
|  | PJH - PSG | -0.39318 | 0.471407 | -0.83406 | 0.918576 |
| K | BRI - CDA | -11.7 | 11.46742 | -1.02028 | 0.844689 |
|  | BRI - QCL | -2.73636 | 11.20377 | -0.24424 | 0.999183 |
|  | BRI - PJH | -17.3727 | 11.20377 | -1.55061 | 0.535995 |
|  | BRI - PSG | -5.475 | 12.16304 | -0.45013 | 0.991226 |
|  | CDA - QCL | 8.963636 | 11.20377 | 0.800055 | 0.929224 |
|  | CDA - PJH | -5.67273 | 11.20377 | -0.50632 | 0.986324 |
|  | CDA - PSG | 6.225 | 12.16304 | 0.511796 | 0.985763 |
|  | QCL - PJH | -14.6364 | 10.93376 | -1.33864 | 0.668963 |
|  | QCL - PSG | -2.73864 | 11.91479 | -0.22985 | 0.999357 |
|  | PJH - PSG | 11.89773 | 11.91479 | 0.998568 | 0.85461 |

**Additional file; Table S5.** Community-wise model statistics describing relationships between Shannon diversity and environmental variables, including sample size (n), coefficient of determination (R²), adjusted R², and residual standard deviation (σ).

| Variable | Community | n | R^2^ | Adj R^2^ | Sigma |
| --- | --- | --- | --- | --- | --- |
| Altitude | BRI | 10 | 0.32312241 | 0.12972881 | 0.42604378 |
|  | CDA | 10 | 0.00628356 | -0.27763542 | 0.56171517 |
|  | QCL | 11 | 0.02136579 | -0.22329276 | 0.18219255 |
|  | PJH | 11 | 0.24122232 | 0.0515279 | 0.25404505 |
|  | PSG | 8 | 0.09815199 | -0.26258721 | 0.41477906 |
| Clay | BRI | 10 | 0.27271325 | 0.06491703 | 0.44162332 |
|  | CDA | 10 | 0.30784797 | 0.11009024 | 0.46879791 |
|  | QCL | 11 | 0.07711417 | -0.15360729 | 0.17692712 |
|  | PJH | 11 | 0.16902842 | -0.03871448 | 0.26585605 |
|  | PSG | 8 | 0.0598671 | -0.09682172 | 0.38659314 |
| Silt | BRI | 10 | 0.1900869 | -0.04131684 | 0.46603482 |
|  | CDA | 10 | 0.0042587 | -0.28023882 | 0.56228717 |
|  | QCL | 11 | 0.49066359 | 0.36332949 | 0.13143856 |
|  | PJH | 11 | 0.37002455 | 0.21253069 | 0.23148096 |
|  | PSG | 8 | 0.10869211 | -0.24783105 | 0.41234812 |
| Sand | BRI | 10 | 0.06779324 | -0.19855155 | 0.49998305 |
|  | CDA | 10 | 0.01235712 | -0.26982656 | 0.55999595 |
|  | QCL | 11 | 0.52998706 | 0.41248382 | 0.12626278 |
|  | PJH | 11 | 0.26824389 | 0.08530486 | 0.24948052 |
|  | PSG | 8 | 0.10615865 | -0.25137788 | 0.41293374 |
| pH | BRI | 10 | 0.34689922 | 0.160299 | 0.41849402 |
|  | CDA | 10 | 0.28054346 | 0.07498445 | 0.47795521 |
|  | QCL | 11 | 0.45774649 | 0.32218311 | 0.13561934 |
|  | PJH | 11 | 0.01509616 | -0.2311298 | 0.28943455 |
|  | PSG | 8 | 0.0984125 | -0.26222251 | 0.41471915 |
| EC | BRI | 10 | 0.02015752 | -0.25979747 | 0.51259845 |
|  | CDA | 10 | 0.2597242 | 0.04821682 | 0.4848213 |
|  | QCL | 11 | 0.15017072 | -0.0622866 | 0.1697799 |
|  | PJH | 11 | 0.35669296 | 0.1958662 | 0.23391744 |
|  | PSG | 8 | 0.12236443 | -0.22868979 | 0.40917327 |
| Tss | BRI | 10 | 0.09685563 | -0.16118562 | 0.49212763 |
|  | CDA | 10 | 0.31453033 | 0.11868186 | 0.46652942 |
|  | QCL | 11 | 0.3680869 | 0.21010862 | 0.14640271 |
|  | PJH | 11 | 0.20149142 | 0.00186428 | 0.2606113 |
|  | PSG | 8 | 0.26068735 | -0.03503771 | 0.37554693 |
| CaCO3 | BRI | 10 | 0.11133027 | -0.14257537 | 0.48816805 |
|  | CDA | 10 | 0.09784247 | -0.15991682 | 0.53521232 |
|  | QCL | 11 | 0.15845045 | -0.05193694 | 0.16895081 |
|  | PJH | 11 | 0.04787054 | -0.19016182 | 0.28457809 |
|  | PSG | 8 | 0.11883994 | -0.23362409 | 0.40999404 |
| OM | BRI | 10 | 0.25245393 | 0.03886934 | 0.447732 |
|  | CDA | 10 | 0.0187431 | -0.26161602 | 0.55818258 |
|  | QCL | 11 | 0.27215659 | 0.09019574 | 0.15712287 |
|  | PJH | 11 | 0.42639109 | 0.28298887 | 0.22088254 |
|  | PSG | 8 | 0.20292653 | -0.11590286 | 0.38994138 |
| N | BRI | 10 | 0.32362644 | 0.13037685 | 0.42588513 |
|  | CDA | 10 | 0.2245072 | 0.00293782 | 0.49621947 |
|  | QCL | 11 | 0.47544692 | 0.34430865 | 0.1333875 |
|  | PJH | 11 | 0.06970094 | -0.16287383 | 0.28129677 |
|  | PSG | 8 | 0.35998775 | 0.10398285 | 0.34941721 |
| P | BRI | 10 | 0.60764922 | 0.495549 | 0.32436682 |
|  | CDA | 10 | 0.19794243 | -0.03121688 | 0.50464699 |
|  | QCL | 11 | 0.12774998 | -0.09031253 | 0.17200494 |
|  | PJH | 11 | 0.0147719 | -0.23153513 | 0.28948219 |
|  | PSG | 8 | 0.09080132 | -0.27287815 | 0.41646599 |
| K | BRI | 10 | 0.21562098 | -0.00848731 | 0.45862967 |
|  | CDA | 10 | 0.03637571 | -0.23894552 | 0.55314474 |
|  | QCL | 11 | 0.0924518 | -0.13443525 | 0.17545077 |
|  | PJH | 11 | 0.35463018 | 0.19328773 | 0.23429217 |
|  | PSG | 8 | 0.20442236 | -0.1138087 | 0.38957531 |

Table S6. Independent contributions of environmental predictors from hierarchical partitioning

| **Model** | **Predictor** | **Independent contribution** | **Contribution (%)** | **p-value** |
| --- | --- | --- | --- | --- |
| Selected CCA model | Sand | 0.0528 | 41.57 | 0.001 |
| Selected CCA model | Clay | 0.0424 | 33.39 | 0.003 |
| Selected CCA model | Slope | 0.0318 | 25.04 | 0.003 |
| Altitude-added model | Sand | 0.0504 | 43.45 | 0.001 |
| Altitude-added model | Clay | 0.0412 | 35.52 | 0.002 |
| Altitude-added model | Slope | 0.0319 | 27.50 | 0.013 |
| Altitude-added model | Altitude | −0.0072 | −6.21 | 0.590 |

Table S7. Beta-deviation null-model test for βSIM turnover

| **Metric** | **Observed** | **Null mean** | **Null SD** | **z-deviation** | **p greater** | **Two-sided p** |
| --- | --- | --- | --- | --- | --- | --- |
| All-pair mean βSIM | 0.757 | 0.765 | 0.008 | −1.050 | 0.822 | 0.358 |
| Within-community mean βSIM | 0.408 | 0.767 | 0.014 | −25.829 | 1.000 | 0.002 |
| Between-community mean βSIM | 0.837 | 0.765 | 0.008 | 8.593 | 0.001 | 0.002 |
| Between-minus-within βSIM | 0.429 | −0.002 | 0.014 | 31.189 | 0.001 | 0.002 |
