# Supplementary material for: Species Turnover and Environmental Filtering Structure Plant Communities in Moist Temperate Forests
Source: Ecol Evol. 2026 Jul 29;16(8):e74054. doi: 10.1002/ece3.74054 (PMC13420373; doi:10.1002/ece3.74054)
Supplement: Supplementary file 2 — Figure S1: Pearson correlation matrix among environmental variables used in the canonical correspondence analysis (CCA). Colors indicate the direction and strength of pairwise correlations, with blue representing positive correlations and red representing negative correlations. Values within cells represent Pearson correlation coefficients (r). Figure S2: Beta‐deviation null‐model test for species turnover among plant communities. Gray bars show the null distribution of between‐minus‐within βSIM values generated by randomizing the occurrence matrix while preserving site richness and species occupancy. The red vertical line represents the observed value. Figure S3: Hierarchical partitioning of independent contributions for the selected CCA predictors. Bars show the independent adjusted R 2 contribution of sand, clay, and slope to community composition. Figure S4: Hierarchical partitioning after adding altitude to the selected CCA predictors. Bars show the independent adjusted R 2 contribution of sand, clay, slope, and altitude. Altitude showed no significant independent contribution. Figure S5: Variance partitioning of Hellinger‐transformed community composition between edaphic and topographic predictor sets. Values represent adjusted R 2 fractions for pure edaphic, pure topographic, shared, and residual components. [file ECE3-16-e74054-s002.docx]

Supplementary figures


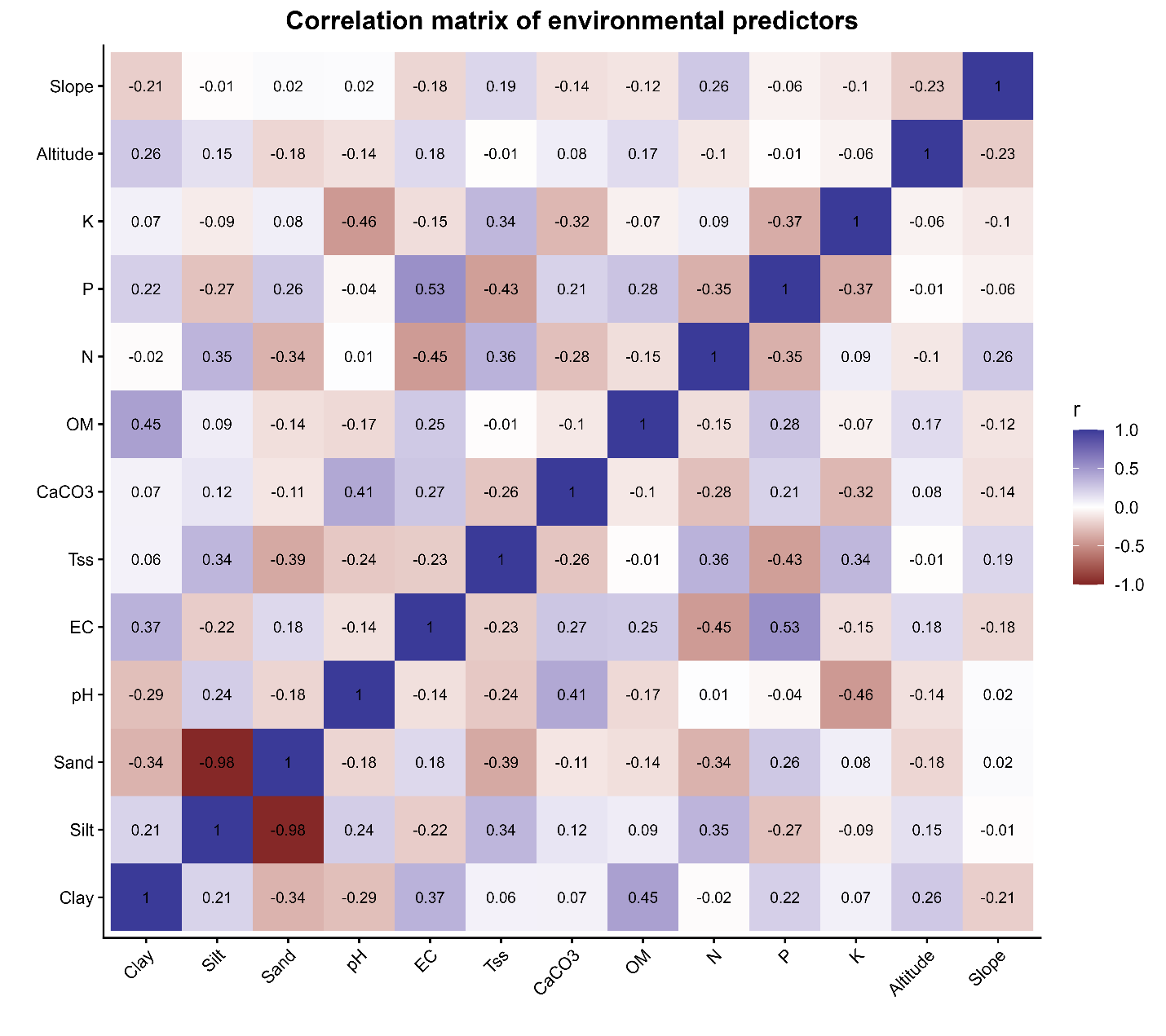


**Figure S1.** Pearson correlation matrix among environmental variables used in the canonical correspondence analysis (CCA). Colors indicate the direction and strength of pairwise correlations, with blue representing positive correlations and red representing negative correlations. Values within cells represent Pearson correlation coefficients (r).

Supplementary figures


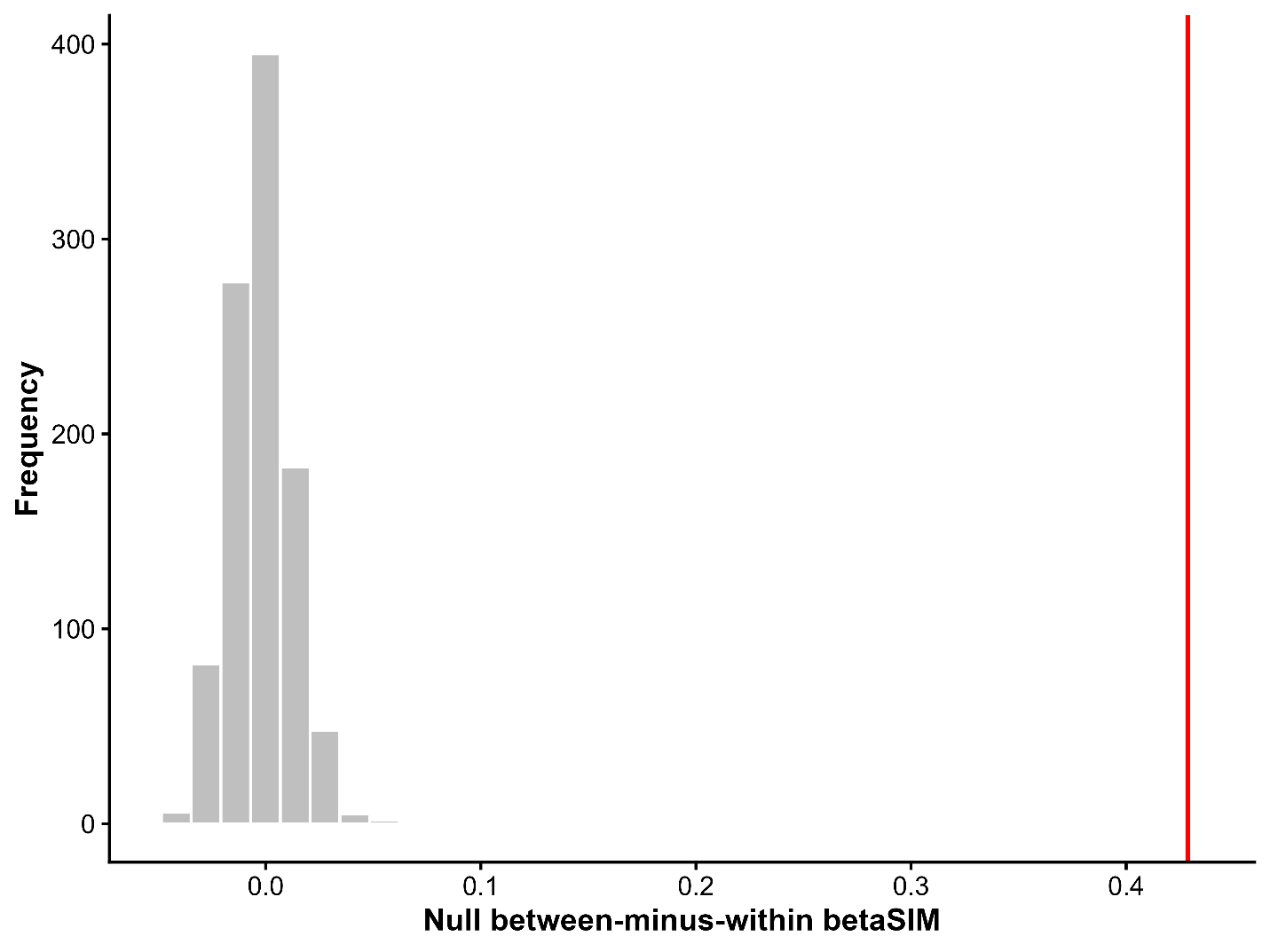


**Figure S2.** Beta-deviation null-model test for species turnover among plant communities. Grey bars show the null distribution of between-minus-within βSIM values generated by randomizing the occurrence matrix while preserving site richness and species occupancy. The red vertical line represents the observed value.


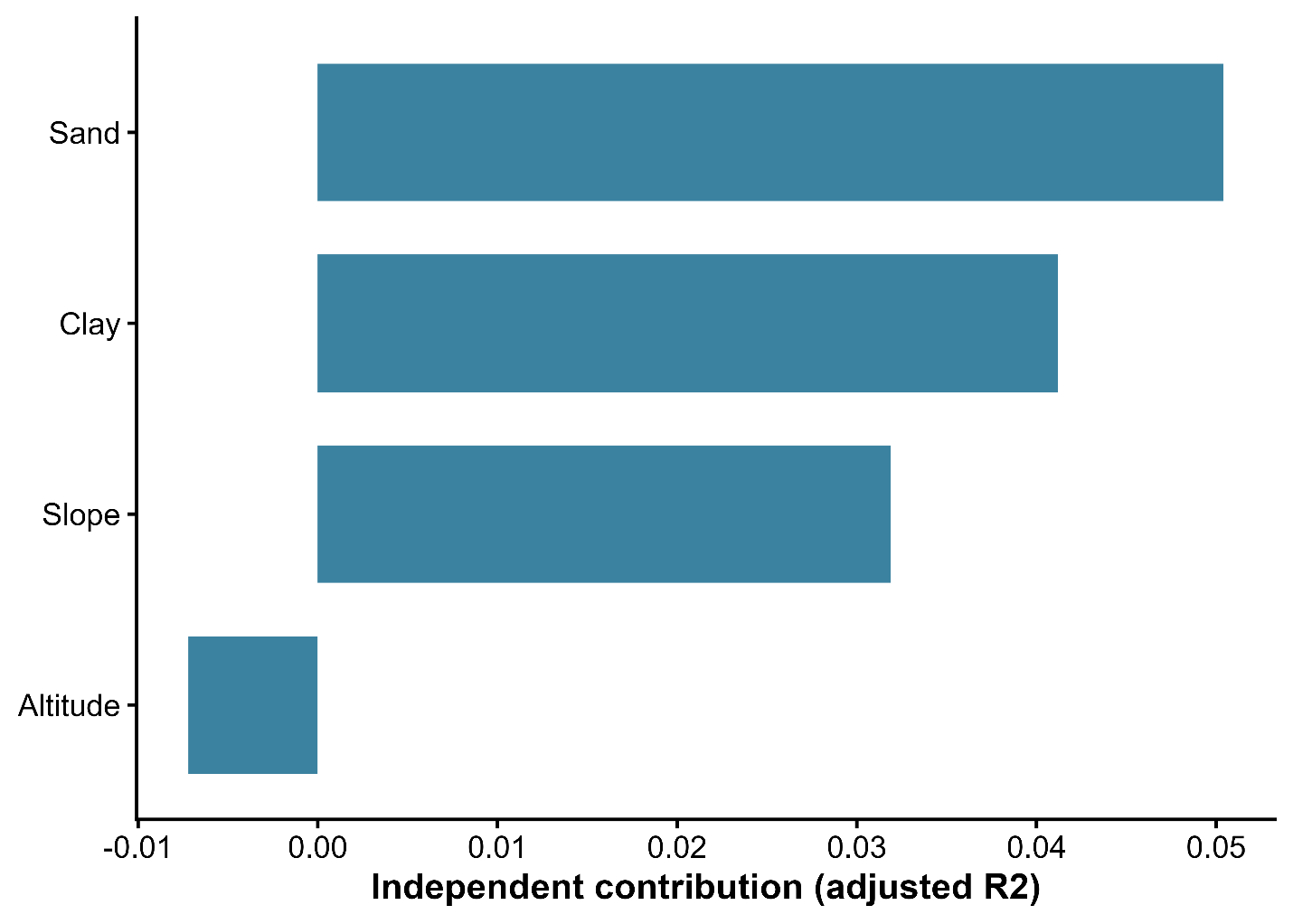


**Figure S3.** Hierarchical partitioning of independent contributions for the selected CCA predictors. Bars show the independent adjusted R² contribution of sand, clay, and slope to community composition.


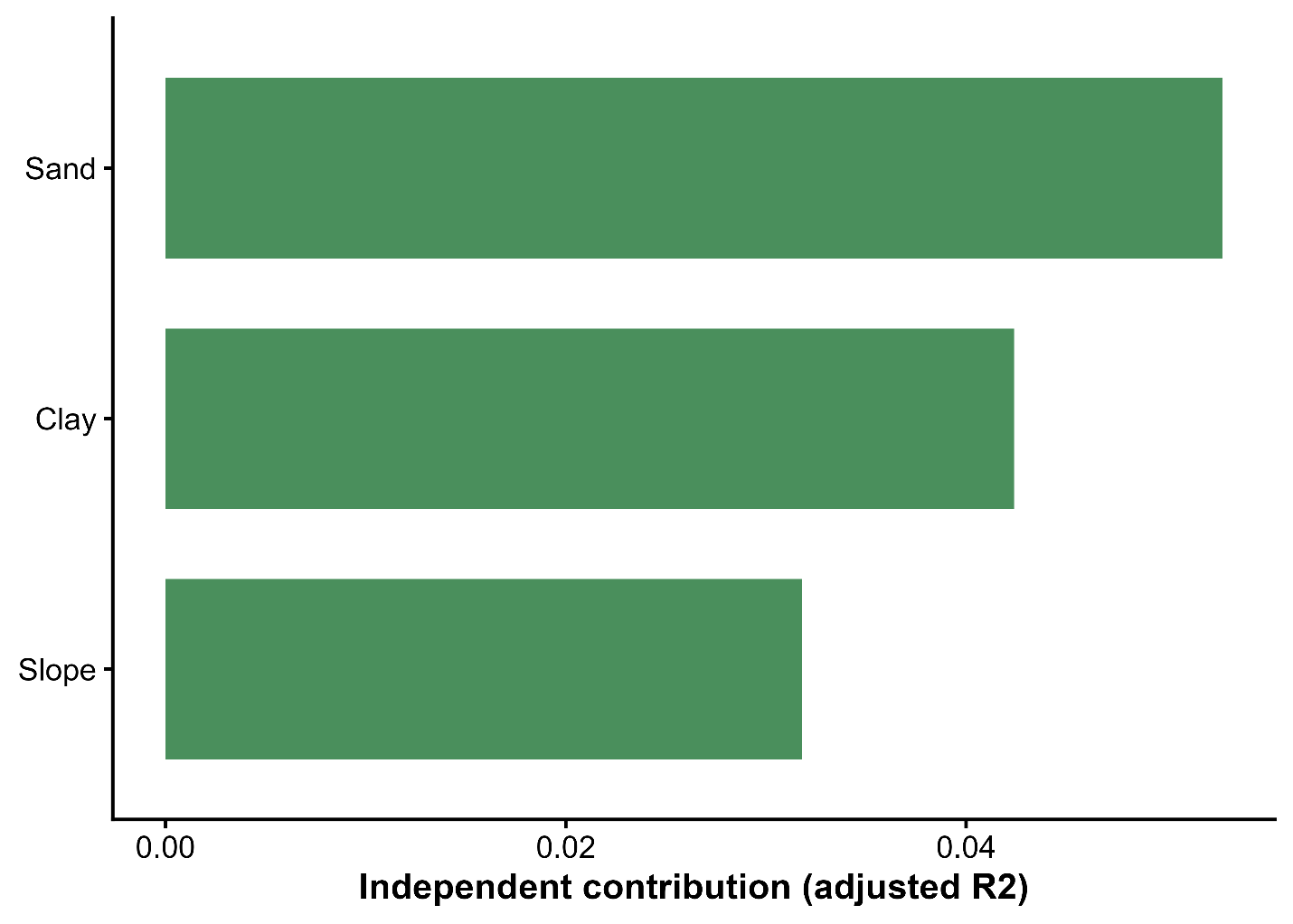


**Figure S4.** Hierarchical partitioning after adding altitude to the selected CCA predictors. Bars show the independent adjusted R² contribution of sand, clay, slope, and altitude. Altitude showed no significant independent contribution.


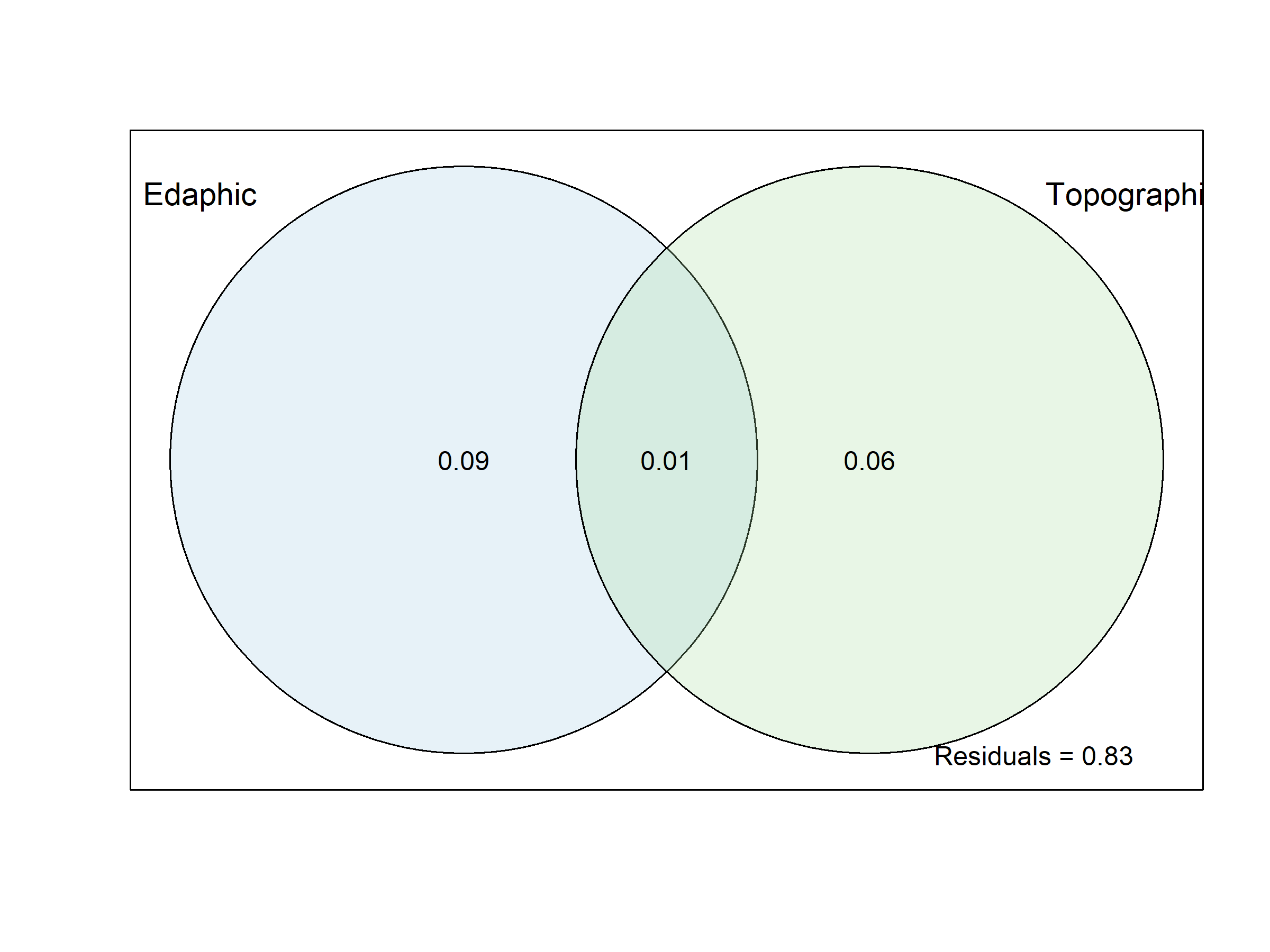


**Figure S5.** Variance partitioning of Hellinger-transformed community composition between edaphic and topographic predictor sets. Values represent adjusted R² fractions for pure edaphic, pure topographic, shared, and residual components.
